# Alterations in the plasma proteome persist ten months after recovery from mild to moderate SARS-CoV-2 infection

**DOI:** 10.3389/fimmu.2024.1448780

**Published:** 2024-09-11

**Authors:** Julio A. Huapaya, Salina Gairhe, Shreya Kanth, Xin Tian, Cumhur Y. Demirkale, David Regenold, Jian Sun, Nicolas F. Lynch, Renjie Luo, Alisa Forsberg, Robin Dewar, Tauseef Rehman, Willy Li, Janell Krack, Janaki Kuruppu, Etsubdink A. Aboye, Christopher Barnett, Jeffrey R. Strich, Richard Davey, Richard Childs, Daniel Chertow, Joseph A. Kovacs, Parizad Torabi-Parizi, Anthony F. Suffredini

**Affiliations:** ^1^ Critical Care Medicine Department, Clinical Center, National Institutes of Health, Bethesda, MD, United States; ^2^ National Heart, Lung, and Blood, Institute, National Institutes of Health, Bethesda, MD, United States; ^3^ Office of Biostatistics Research, National Heart, Lung, and Blood, Institute, National Institutes of Health, Bethesda, MD, United States; ^4^ Laboratory of Immunoregulation, National Institute of Allergy and Infectious Diseases, National Institutes of Health, Bethesda, MD, United States; ^5^ National Institute of Allergy and Infectious Diseases (NIAID) Collaborative Bioinformatics Resource, National Institute of Allergy and Infectious Diseases, National Institutes of Health, Bethesda, MD, United States; ^6^ Department of Statistics, The George Washington University, Washington, DC, United States; ^7^ Virus Isolation and Serology Laboratory, Applied and Developmental Directorate, Frederick National Laboratory, Frederick, MD, United States; ^8^ Pharmacy Department, Clinical Center, National Institutes of Health, Bethesda, MD, United States; ^9^ Medstar Heart and Vascular Institute, Medstar Washington Hospital Center, Washington, DC, United States; ^10^ Division of Cardiology, University of California, San Francisco, CA, United States; ^11^ Laboratory of Transplantation Immunotherapy, National Heart Lung and Blood Institute, National Institutes of Health, Bethesda, MD, United States

**Keywords:** SARS-CoV-2, proteomics, vaccination, breakthrough infections, post-acute sequelae, inflammation

## Abstract

**Background:**

Limited data are available describing the effects of SARS-CoV-2 breakthrough infections on the plasma proteome.

**Methods:**

PCR-positive SARS-CoV-2 patients, enrolled in a natural history study, underwent analysis of the plasma proteome. A prospective cohort of 66 unvaccinated and 24 vaccinated persons with different degrees of infection severity were evaluated acutely (within 40 days of symptom onset), and at three and ten months. Comparisons based on vaccination status alone and unsupervised hierarchical clustering were performed. A second cohort of vaccinated Omicron patients were evaluated acutely and at ten months.

**Results:**

Acutely, unvaccinated patients manifested overexpression of proteins involved in immune and inflammatory responses, while vaccinated patients exhibited adaptive immune responses without significant inflammation. At three and ten months, only unvaccinated patients had diminished but sustained inflammatory (C3b, CCL15, IL17RE) and immune responses (DEFA5,TREM1). Both groups had underexpression of pathways essential for cellular function, signaling, and angiogenesis (AKT1, MAPK14, HSPB1) across phases. Unsupervised clustering, based on protein expression, identified four groups of patients with variable vaccination rates demonstrating that additional clinical factors influence the plasma proteome. The proteome of vaccinated Omicron patients did not differ from vaccinated pre-Omicron patients.

**Conclusions:**

Vaccination attenuates the inflammatory response to SARS-CoV-2 infection across phases. However, at ten months after symptom onset, changes in the plasma proteome persist in both vaccinated and unvaccinated individuals, which may be relevant to post-acute sequelae of SARS-CoV-2 and other viral infections associated with post-acute infection syndromes.

## Introduction

The global impact of SARS-CoV-2 has been substantial, affecting millions of persons worldwide (1). To gain a deeper understanding of the host-virus response and identify molecular signatures associated with disease progression, high density proteomic methods have emerged as valuable tools (2–12). These studies have provided rich data, allowing for the exploration of pathways related to inflammation, coagulation, extracellular matrix organization (matrisome), complement activation, and other pathways implicated in pathogenesis (5, 13, 14). The duration and resolution of blood proteomic perturbation following SARS-CoV-2 infection varies with some reports describing normalization within two weeks (6) and others reporting persistent changes for months following infection (5, 7). These differences in part reflect clinical heterogeneity among SARS-CoV-2 patients.

Investigating the plasma proteome during and post-SARS-CoV-2 infection is important as it reflects systemic responses and can reveal biomarkers and pathways linked to disease severity and recovery. The impact of vaccination on the host proteomic responses during acute breakthrough infections and recovery has not been well delineated. We have previously reported that vaccinated patients with breakthrough infections have more favorable cellular immune responses with reduced inflammation compared with unvaccinated patients (15). However, vaccinated patients who experience breakthrough infections remain at increased risk for death and post-acute sequela of COVID compared to vaccinated patients who do not experience breakthrough infections (16). Here, we evaluated the alterations in the plasma proteome responses in a prospective cohort of vaccinated and unvaccinated patients compared with healthy controls during the acute illness and at three and ten months post-infection. These findings have implications for understanding the post-acute sequelae of SARS-CoV-2 as well as other viral infections associated with post-acute infection syndromes (17).

## Materials and methods

### Study cohort

Ninety hospitalized and ambulatory patients who were SARS-CoV-2 PCR test positive were enrolled in a natural history study of SARS-CoV-2 from May 2020 to June 2022 at two centers in Maryland and Washington, D.C. (NCT04401449). Patients were enrolled during the acute phase of infection (within 40 days of symptom onset). A subset of patients was then followed longitudinally with in-person visits (at three and ten months) and telephone follow-ups (every 2-4 weeks). Patients had high resolution chest computed tomography (CT) and a thorough interview performed at each in-person visit. The presence or absence of symptoms was assessed at each telephone follow-up. The full list of symptoms is available in **Supplementary Information** (text description, page 4). A reference cohort of 20 healthy controls (HC), 13 who were vaccinated and without infection, were included in the analysis. Additionally, a second cohort of vaccinated Omicron patients with data acutely and at ten months were evaluated. The study was approved by the NIH institutional review board and all patients provided written consents for their study participation.

Clinical severity of SARS-CoV-2 patients was determined using a modified NIAID ordinal scale (OS) score at two data points: highest OS, and OS at the time of sample acquisition. Mild infections were defined as patients who did not require oxygen during their illness (OS ≤ 4). Moderate infections included patients who required ≤ 6 liters/minute of oxygen (OS of 5). Severe disease included patients who required supplemental oxygen by non-rebreather mask, high-flow nasal cannula, or mechanical ventilatory support (OS ≥ 6). Full vaccination was defined as having received at least two doses of the BNT162b2 (Pfizer/BioNTech) or mRNA-1273 (Moderna) vaccines or a single dose of the Ad26.COV2.S (Johnson & Johnson) vaccine. The Charlson comorbidity score were calculated for each patient. Antibodies to specific variant receptor binding domains were measured. Detailed methods are in the **Supplementary Information** (text description, page 2*)*.

### Protein measurements

Blood was collected in EDTA-containing vacutainers, centrifuged, and the plasma separated and frozen until analysis. The plasma was then analyzed in three batches using the modified -aptamer-based array (SOMAScan Assay v4.1, Boulder, CO) that targets 7596 proteins. Aptamers that are not specific to human proteins were excluded (n=308) resulting in 7288 human proteins that were included in the analyses. Given that certain proteins had more than one aptamer recognize different amino acid sequences on the targeted protein, only unique SOMAmers were included in the enrichment pathway analysis. Quality analytic measures are described in the **Supplementary Information** (text description, page 3).

### Statistical analysis

Data were presented as means, standard deviations (SD), medians, and percentiles. We first performed analyses based on vaccination status on all the initial samples of SARS-CoV-2 patients. We then performed an Uniform Manifold Approximation and Projection (UMAP) analysis and unsupervised hierarchical clustering analysis using the Ward method (18) to identify patients with similar protein expression patterns. Vaccinated, unvaccinated, and each of the SARS-CoV-2 groups, identified via hierarchical clustering, were directly compared to healthy controls. The same analysis was applied for the patients with a repeat sample at three months post-infection. For these comparisons, we used the full SOMAScan assay v4.1. For the comparison between the samples of SARS-CoV-2 patients ten months post-infection and healthy controls, we used a customized panel composed of 1500 proteins that we identified when comparing G1-G4 SARS-CoV-2 patients in the acute phase versus healthy controls in the current study. Further, vaccinated patients were also analyzed according to the most common circulating variant at the time of infection (pre-Omicron vs Omicron). For each protein biomarker at a given phase post SARS-CoV-2 infection, the analysis of covariance (ANCOVA) model was used to compare the mean levels of biomarkers between patient groups and healthy controls, adjusted for patient’s age, sex, race and time from symptom onset when appropriate. Because biomarker values could be very skewed even after a log-transformation, the rank-based inverse normal transformation was applied to the biomarker values in the models (19). To control for multiple testing, the p-values for the individual biomarkers were adjusted using the Benjamini-Hochberg false discovery rate procedure. A false discovery rate (FDR)<1% and fold change (FC)>1.25 was considered significant. Protein networks and pathway analysis associated with the different groups were analyzed using Metascape (metascape.org) (20), and QIAGEN Ingenuity Pathway Analysis (IPA) (QIAGEN Inc) (21). Overexpressed and underexpressed proteins were analyzed separately. In IPA, we used the graphical summary option which applies machine learning techniques to provide an overview of the major biological themes of the data by including canonical pathways, upstream regulators, diseases, and biological functions. In an exploratory fashion, we used a statistical machine learning approach to select the most influential covariates for persistent symptoms and radiological abnormalities at ten months post-infection. Persistent symptoms were further classified by four systems (cardiopulmonary, neurologic, musculoskeletal, and other). For each outcome, we used random forest method to provide variable importance ranking based on the mean decrease in Gini index for all the protein biomarkers from SOMAScan and patients’ baseline characteristics (age, sex, race, and vaccination status). Then, we manually selected 11-12 of the most relevant proteins among the top 30, as well as a smaller group of 5 proteins to build up a CART model to predict symptoms at ten months. Lastly, we used linear mixed with both random intercept and slopes to assess the time trend of the proteins that accounted for within-subject correlation and to estimate two subject-specific slopes (b1 and b2) to measure the changes from acute to three months follow-up and from three months follow-up to ten months follow-up, respectively. Each biomarker was transformed by rank-based inverse normal transformation, and then p-value for estimated b1 and b2 were adjusted over 1500 biomarkers with FDR methods. FDR adjusted p< 0.01 for b0 or b1 was used to select biomarkers with significant change over time. Age, sex, and race were included in the model for all the patients. Further, we provide an online tool that allows visualization of comparisons for protein biomarkers between vaccinated and unvaccinated patients and patient clusters, as well as the longitudinal trends over time (https://dir.nhlbi.nih.gov/lab/suffredini/proteomics/). Graphical summary was created with Biorender.com.

## Results

### Vaccination status drives plasma proteome during acute SARS-CoV-2 infection

**Figure 1:**
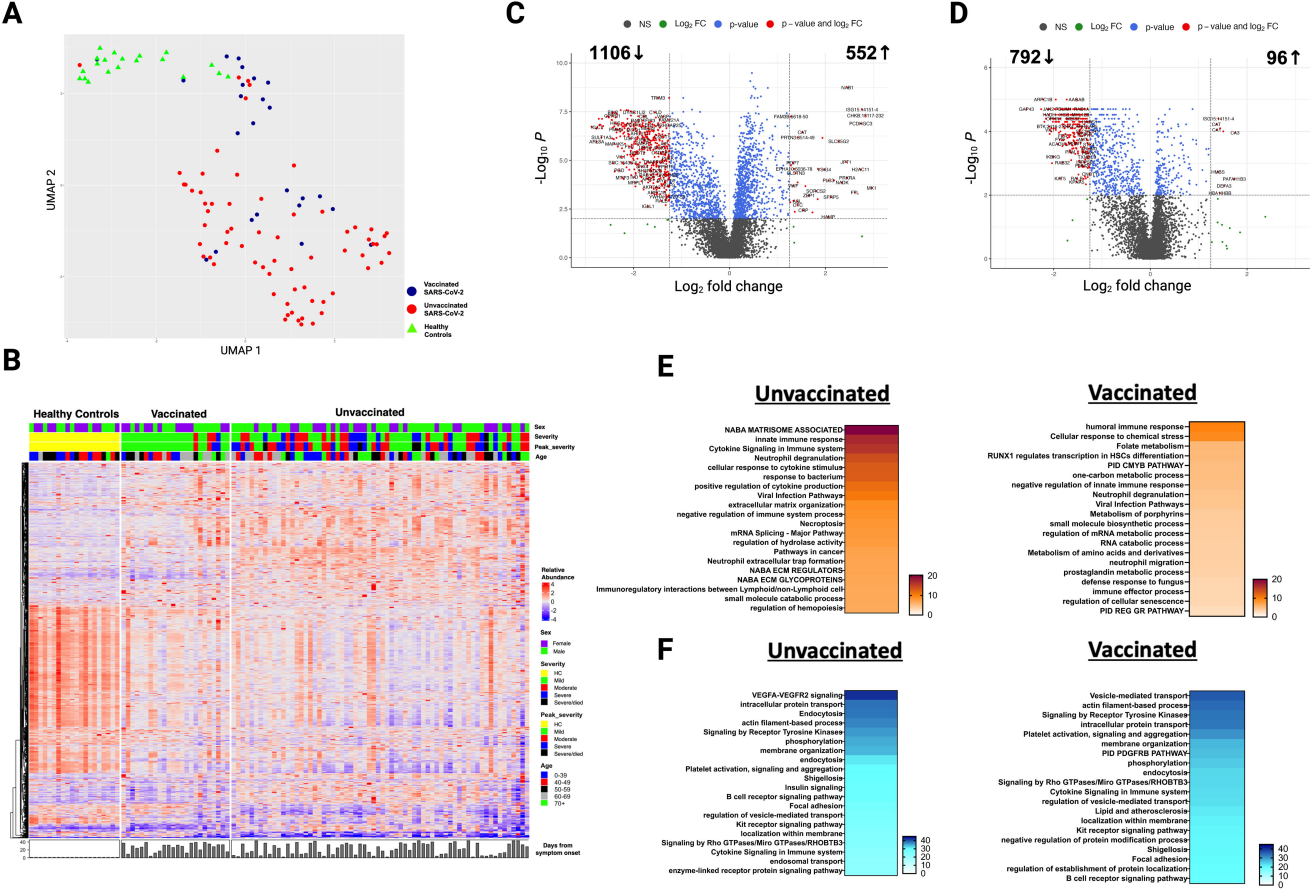
Alterations in the plasma proteome in vaccinated and unvaccinated SARS-CoV-2 patients during the acute phase of infection. **(A)** Uniform manifold approximation and projection (UMAP) of acute changes in the plasma proteome from 90 SARS-CoV-2 patients and 20 healthy controls. Blue circles – vaccinated, red circles – unvaccinated, and green triangles – healthy controls. **(B)** Heatmap of differentially expressed proteins (DEP) in vaccinated and unvaccinated SARS-CoV-2 patients compared to healthy controls. Each row represents a single protein; each column represents a patient. Volcano plots of DEP in plasma comparing **(C)** unvaccinated SARS-CoV-2 patients vs healthy controls and **(D)** vaccinated SARS-CoV-2 patients vs healthy controls. In **(C, D)**, the horizontal dashed line denotes a cutoff of 0.01 for the FDR corrected p value after age, sex, and race adjustments. Vertical dashed line denotes a cutoff of 1.25 for the fold change. Pathway enrichment analysis of overexpressed proteins in **(E)** SARS-CoV-2 patients (according to vaccination status) vs healthy controls and of **(F)** underexpressed proteins in SARS-CoV-2 patients (according to vaccination) vs healthy controls. Gradation of colors in **(E, F)** reflect the -log 10 (*p*) value which indicates the statistically enriched terms using Metascape. An on-line summary of each protein across the different conditions analyzed is available at https://dir.nhlbi.nih.gov/lab/suffredini/proteomics/.

Baseline characteristics of 66 unvaccinated and 24 vaccinated SARS-CoV-2 patients, and 20 HC are described in **Table 1**. Analysis based on vaccination status using plasma collected during acute infection indicate that unvaccinated and vaccinated SARS-CoV-2 patients cluster separately from each other and from HC (**Figure 1A**). After adjusting for age, sex, and race, we observed that 1839 unique proteins were differentially expressed in unvaccinated and vaccinated SARS-CoV-2 patients compared with HC (**Figures 1B-D**).

**Table 1:** Characteristics of SARS-CoV-2 patients at various phases of infection.

Characteristic	All SARS-CoV-2 – Initial Sampling		Three months follow-up		Ten months follow-up	
Group	Unvaccinated (n=66)	Vaccinated^a^ (n=24)	Unvaccinated (n=22)	Vaccinated (n=10)	Unvaccinated (n=24)	Vaccinated (n=10)
**Age (years)**	56 (44-66)	58.5 (43-66)	47 (32-62)	49 (40-60)	49 (31-63)	49 (40-60)
**Sex (female)**	26 (39%)	12 (50%)	6 (27.3%)	6 (60%)	8 (33.3%)	6 (60%)
Race-Ethnicity						
White Black Asian Latino	14 (52%) 34 (21%) 3 (5%) 15 (23%)	11 (46%) 9 (38%) 2 (8%) 2 (8%)	7 (31.8%) 4 (18.2%) 3 (13.6%) 8 (36.4%)	6 (60%) 1 (10%) 2 (20%) 1 (10%)	9 (37.5%) 4 (15.4%) 3 (11.5%) 8 (33.3%)	6 (60%) 1 (10%) 2 (20%) 1 (10%)
Vaccine						
BNT162b2 mRNA-1273 Ad26.COV2.S	0^b^ 0^c^ 0	12 (50%) 9 (38%) 3 (12%)	3 (14%) 1^e^ (5%) -	7 (70%) 1 (10%) 2 (20%)	14 (58%) 5 (21%) -	7 (70%) 1 (10%) 2 (20%)
**Time (days) of sample after symptoms onset**	12 (7 – 26)	17 (7 – 39)	85 (80-89)	81 (70-85)	284 (271-317)	281 (268-301)
**Hospitalized**	57 (86%)	10 (42%)	–	–	–	–
**Time (days) from full vaccination to sample**	–	175 (129 – 329)	-	204 [172-255]	150 [118-233]	420 [399-461]
**Time (days) from last vaccine to sample** **^d^**	–	137 (99 – 174)	-	196 [164-244]	133 [73-181]	140 [77-184]
NIAID Highest Ordinal scale						
4 (no oxygen) 5 (low-flow oxygen 6 (high-flow oxygen) 7 (MV/death)	19 (29%) 23 (35%) 19 (29%) 5 (7%)	20 (83%) 2 (8%) 2 (8%) -	11 (50%) 3 (13.6%) 8 (36.4%) -	10 (100%) - - -	13 (54.2%) 2 (8.3%) 9 (37.5%) -	10 (100%) - - -
NIAID Ordinal scale at time of sample						
4 (no oxygen) 5 (low-flow oxygen 6 (high-flow oxygen) 7 (MV/death)	33 (50%) 19 (29%) 13 (20%) 1 (1%)	20 (83%) 3 (13%) 1 (4%) -	20 (90.9%) 2 (9.1%) - -	10 (100%) - - -	22 (91.7%) 2 (8.3%) - –	10 (100%) - - -
SARS-CoV-2 variants						
Wuhan Alpha Delta Gamma Mu Omicron BA.1 other	13 (20%) 13 (20%) 25 (38%) 13 (20%) - - 2 (3%)	9 (38%) 1 (4%) 9 (38%) 2 (8%) 2 (8%) 1 (4%) -	4 (13%) 5 (16%) 7 (22%) 6 (19%) - - -	6 (19%) 1 (3%) 2 (6%) 1 (3%) - - -	4 (12%) 4 (12%) 8 (24%) 8 (24%) - - -	6 (19%) 1 (3%) 2 (6%) 1 (3%) - - -
**Charlson Comorbidity Score**	2 (0 – 3)	2 (0.8 – 5.3)	0 (0-2)	0.5 (0-1.3)	0 (0-2)	0.5 (0-1.3)
**Any SARS-CoV-2 treatment**	49 (74%)	9 (38%)	–	–	–	–
**Any Steroids**	40 (60%)	6 (25%)	–	–	–	–
**Reinfections**	–	–	–	–	0	1

Data are summarized by median (interquartile range) or n(%).

a Five patients (21%) had chronic kidney disease, two receiving immunosuppressive therapy for kidney transplantation.

b 3 patients received only one dose of BNT162b2 (3 days, 1 week, and 7 months before symptoms, respectively).

c 1 patient received only one dose of mRNA-1273 (4 months before symptoms).

d 9 patients received a booster of whom data was available in 8.

e 1 other patient received one dose but was not fully vaccinated at the time of follow-up.

**Figure 2:**
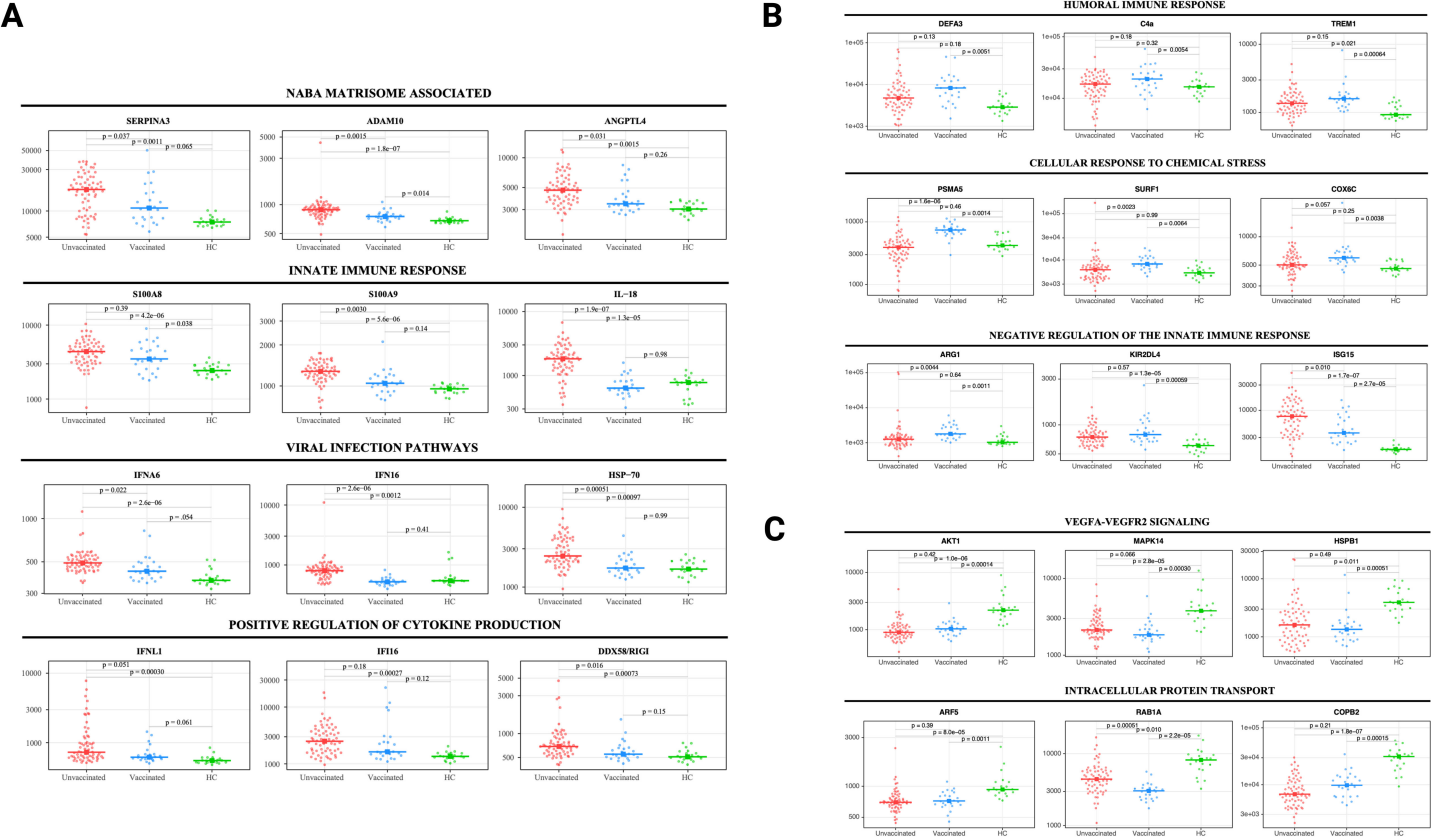
Selected protein levels related to core biological processes differ according to vaccination status during acute infection. Beeswarm plots displaying biological processes related to **(A)** NABA matrisome associated, innate immune response, viral infection, and positive regulation of cytokine production, **(B)** humoral immune response, cellular response to chemical stress, and negative regulation of the innate immune response, and **(C)** VEGFA-VEGFR2 signaling and intracellular protein transport. Protein levels are measured in relative fluorescence units (RFU). For the comparison between unvaccinated and vaccinated SARS-CoV-2 patients, an analysis of covariance model over the rank-based inverse normal transformed biomarkers was used after adjusting for age, sex, race, and days from symptom onset. For the comparison between vaccinated or unvaccinated patients and healthy controls, adjustments were made for age, sex, and race. FDR *p* value (adjusted over 7288 proteins) is shown for each pairwise comparison. An on-line summary of each protein across the different conditions analyzed is available at https://dir.nhlbi.nih.gov/lab/suffredini/proteomics/.

In the acute phase, 552 proteins were overexpressed in unvaccinated patients compared to HC, including proteins involved in critical pathways such as matrisome associated, innate immune response activation, cytokine signaling in the immune system, positive regulation of cytokine production, and viral infection (**Figures 1E**, **2A**). Among vaccinated patients, 96 proteins were overexpressed compared to HC, predominantly involving pathways associated with humoral immune response, cellular response to chemical stress, and the negative regulation of immune response (**Figures 1E**, **2B**). Pathway analyses of overexpressed proteins limited to SARS-CoV-2 patients with samples collected within 30 days from symptoms onset compared with HC yielded similar results to analyses that included all acute SARS-CoV-2 patients (**Supplementary Figure S1**).

While both unvaccinated and vaccinated patients shared enrichment of certain pathways, including viral infection and neutrophil degranulation (**Figure 1E**), the number of proteins and the specific proteins within these pathways significantly varied between groups. Within the neutrophil degranulation pathway, unvaccinated patients exhibited 39 differentially expressed proteins (DEP) compared to HC, including a subset of proteins that were exclusively expressed in this group (SERPINA3, ADAM10, C3, FCAR, MMP9, S100A8, S100A9, and S100A12), while vaccinated patients had only nine DEP compared to HC, including three proteins that were only present in this group (ARG1, GM2A, and PSMA5).

Ingenuity pathway analysis (IPA) also showed several differences between SARS-CoV-2 patients and HC. The graphical summary for both unvaccinated and vaccinated patients exhibited proteins involved in cell movement (neutrophils, leukocytes, myeloid cells, granulocytes, and phagocytes), chemotaxis, engulfment, and killing of bacteria (**Supplementary Figure S2**). However, only unvaccinated patients had proteins involved in infections (e.g. retroviridae, RNA virus, lentivirus, and HIV), hypercytokinemia in the pathogenesis of influenza, and pathogen induced cytokine storm signaling pathway. Similarly, while TNF was included as a regulator in both groups, IL-18, IFNG, IFNA8, IFNA16, IFNL1, and RIGI were only seen in unvaccinated patients. More information on the top canonical pathways for each group is available in **Supplementary Information** (text description, page 5).

In adjusted analyses, we observed 1106 underexpressed proteins in unvaccinated patients and 792 in vaccinated patients compared with HC. Despite the numerical difference, there was a considerable overlap in the pathways affected which include critical biological processes including VEGF-VEGFR2 signaling, vital in angiogenesis and vascular permeability regulation; intracellular protein transport, essential for cellular function and homeostasis; endocytosis, pivotal in cell signaling and nutrient uptake; actin filament-based processes, crucial for cellular structure and movement; and signaling by receptor tyrosine kinases, important in cellular signaling cascades (**Figures 1F**, **2C**). The underexpression of proteins in these pathways among both unvaccinated and vaccinated patients compared with HC indicates potential disruptions and abnormal regulation in key cellular processes in response to the SARS-CoV-2 infection. IPA supported these observations (**Supplementary Figure S3**).

### Persistent proteome abnormalities predominate in unvaccinated SARS-CoV-2 patients at three months post-infection

**Figure 3:**
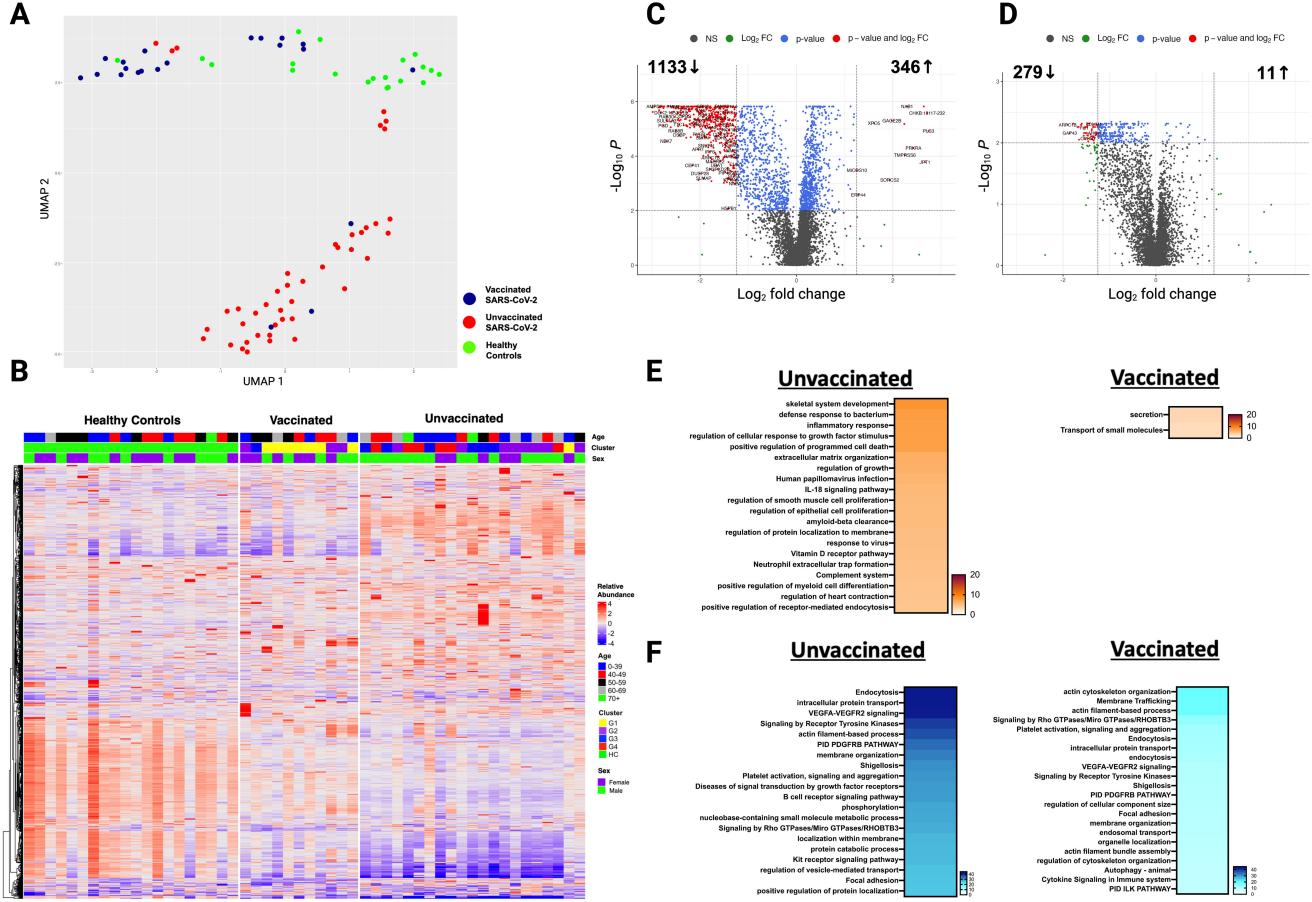
Persistent differences in the plasma proteome of unvaccinated and vaccinated SARS-CoV-2 patients detected at three months post-infection. **(A)** UMAP of the plasma proteome from 32 SARS-CoV-2 patients and 20 healthy controls at three months post-infection. Blue circles - vaccinated patients, red circles -unvaccinated patients, and green circles - healthy controls. **(B)** Heatmap showing DEP between SARS-CoV-2 patients (according to vaccination) and healthy controls at three months post-infection. Each row represents a single protein; each column represents a patient. Volcano plot of DEP in the plasma proteome between **(C)** unvaccinated SARS-CoV-2 patients vs healthy controls at three months post-infection and **(D)** vaccinated SARS-CoV-2 patients vs healthy controls at three months post-infection. Horizontal dashed line denotes a cutoff of 0.01 for the FDR corrected *p* value after age, sex, and race adjustments. Vertical dashed line denotes a cutoff of 1.25 for the fold change. Pathway enrichment analysis of **(E)** overexpressed proteins and **(F)** underexpressed proteins in SARS-CoV-2 patients (according to vaccination status) vs healthy controls at three months post-infection. Gradation of colors reflect the -log10 (*p*) value which indicates the statistically enriched terms using Metascape. An on-line summary of each protein across the different conditions analyzed is available at https://dir.nhlbi.nih.gov/lab/suffredini/proteomics/.

Thirty-two SARS-CoV-2 patients (22 unvaccinated and 10 vaccinated) had a follow-up sample three months post-infection and were compared to HC (**Table 1**). Most unvaccinated SARS-CoV-2 patients clustered separately from vaccinated patients and HC at three months (**Figure 3A**). After adjusting for age, sex, and race, 1333 unique proteins were differentially expressed in vaccinated and unvaccinated SARS-CoV-2 compared with HC (**Figures 3B-D**).

**Figure 4:**
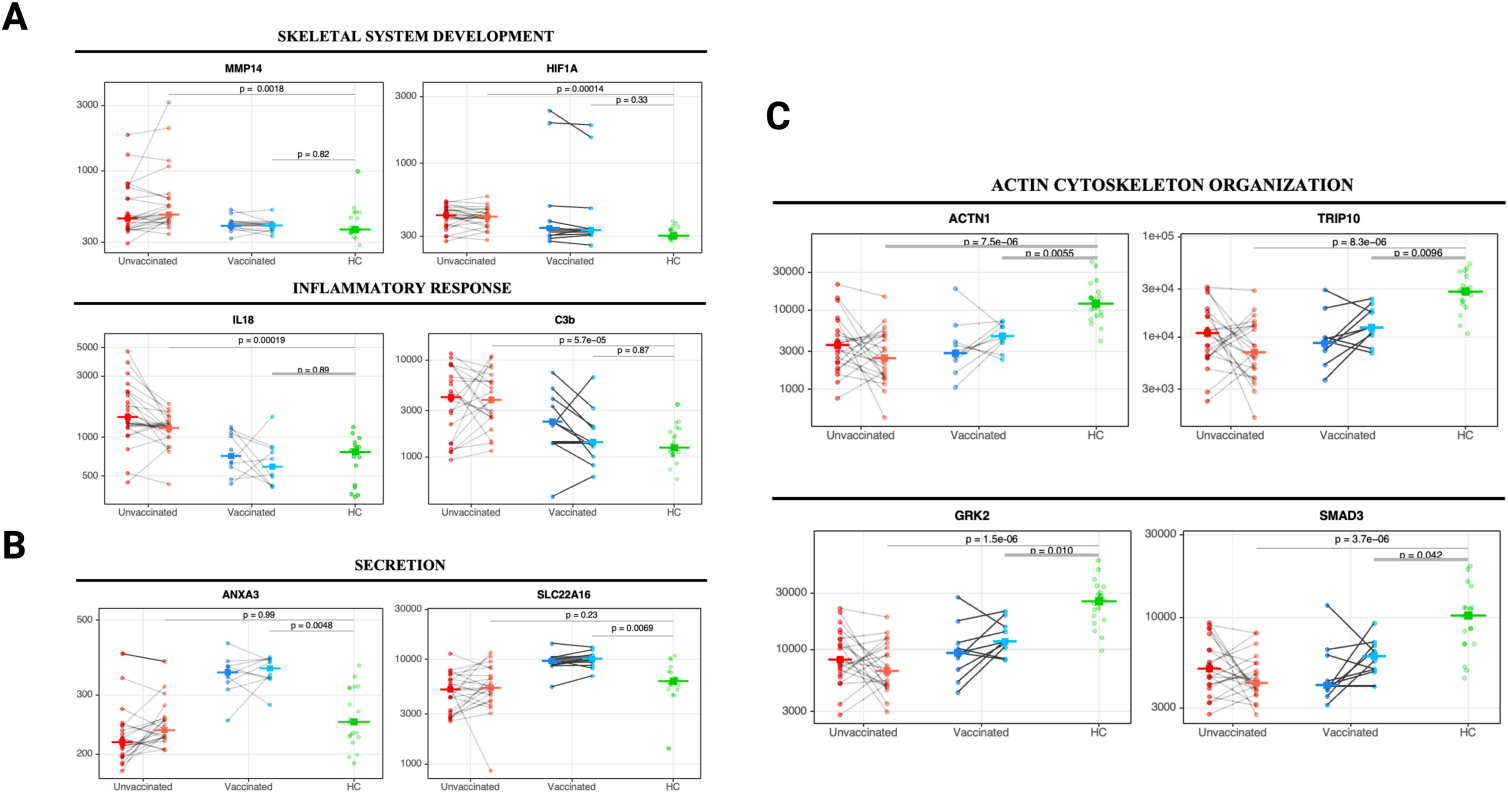
Changes in core biological processes proteins detected in unvaccinated and vaccinated patients between acute infection and three months post-infection. Paired line plots displaying changes in selected DEP from acute infection to three months post-infection in unvaccinated and vaccinated SARS-CoV-2 patients compared with healthy controls. Biological processes related to **(A)** skeletal system development and inflammatory responses, **(B)** cell secretion, and **(C)** actin cytoskeleton organization and endocytosis. Protein levels are measured in relative fluorescence units (RFU). For the comparison between unvaccinated and vaccinated SARS-CoV-2 patients, an analysis of covariance model over the rank-based inverse normal transformed biomarkers was used after adjusting for age, sex, race, and days from symptom onset. For the comparison between vaccinated or unvaccinated patients and healthy controls, adjustments were made for age, sex, and race. FDR *p* value (adjusted over 7288 proteins) is shown for each pairwise comparison. An on-line summary of each protein across the different conditions analyzed is available at https://dir.nhlbi.nih.gov/lab/suffredini/proteomics/.

At three months post-infection, unvaccinated patients had 346 overexpressed proteins contributing to critical pathways including innate immune response, inflammatory response, neutrophil degranulation, and the negative regulation of the immune system process, and skeletal system development compared with HC (**Figures 3E**, **4A**). Similarly, the graphical summary using IPA demonstrated enrichment in several pathways including stimulation of T lymphocytes, immune response of leukocytes, macrophage classical activation signaling pathway, accumulation of leukocytes, antimicrobial response, and cytotoxicity of leukocytes (**Supplementary Figure S4**). Similar to the acute phase, only unvaccinated patients had enrichment of IL-18, IFNG, and TNF. Among vaccinated patients only 11 proteins were overexpressed, primarily contributing to pathways related to the *secretion* and transport of small molecules (**Figures 3E**, **4B**) with no enrichment detected by IPA in this group.

At three months post-infection, we observed 1133 and 279 underexpressed proteins in unvaccinated and vaccinated patients, respectively, compared with HC (**Figures 3F**, **4C**). Both groups shared certain pathways including *VEGF-VEGFR2* signaling, intracellular protein transport, endocytosis, and actin filament-based processes. However, the number and the type of proteins within these pathways differed by group (**Figures 3F**). IPA findings supported these observations (**Supplementary Figure S5**).

### Persistent abnormalities in the plasma proteome of SARS-CoV-2 patients ten months post-infection

**Figure 5:**
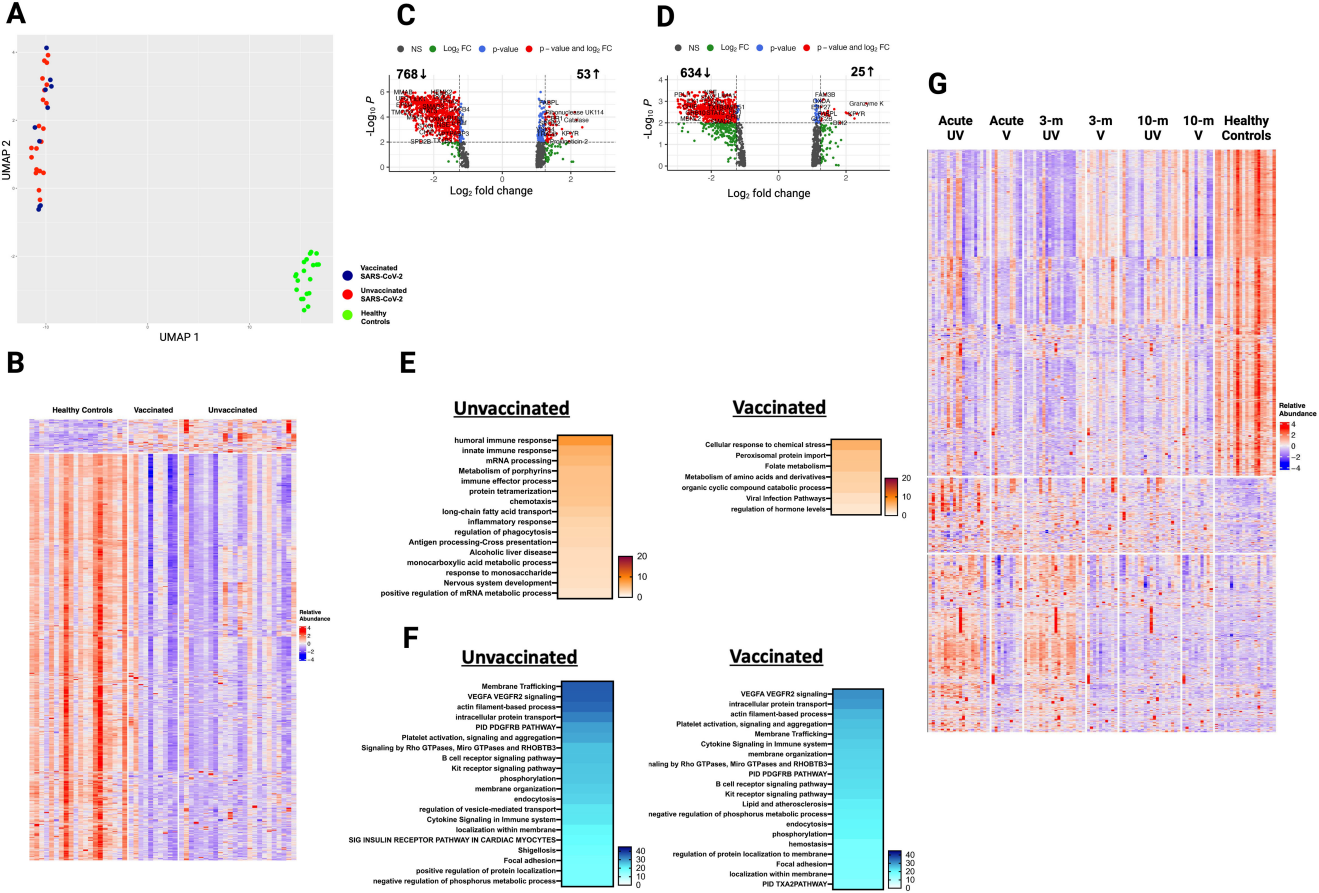
Plasma proteome in vaccinated and unvaccinated SARS-CoV-2 patients at ten- month post-infection. **(A)** UMAP of plasma proteome from 32 SARS-CoV-2 patients and 20 healthy controls using a focused aptamer-assay based on 1500 proteins previously identified DEP in acute phases of SARS-CoV-2 infection. Blue circles - vaccinated patients, red circles - unvaccinated patients, and green triangles - healthy controls. **(B)** Heatmap showing DEP between SARS-CoV-2 patients (according to vaccination) and healthy controls at ten months post-infection. Each row represents a single protein; each column represents a patient. Volcano plot of DEP in plasma between **(C)** unvaccinated SARS-CoV-2 patients vs healthy controls and **(D)** vaccinated SARS-CoV-2 patients vs healthy controls. Horizontal dashed line denotes a cutoff of 0.01 for the FDR corrected *p* value after age, sex, and race adjustments. Vertical dashed line denotes a cutoff of 1.25 for the fold change. Pathway enrichment analysis of **(E)** overexpressed and **(F)** underexpressed proteins in SARS-CoV-2 patients (according to vaccination) vs healthy controls. Colors reflect the -log 10 (*p*) value which indicates the statistically enriched terms using Metascape. **(G)** Heatmap showing all 1500 proteins over the 3 phases of illness and healthy controls. UV - unvaccinated patients and V - vaccinated patients. Each row represents a single protein; each column represents a patient. An on-line summary of each protein across the different conditions analyzed is available at https://dir.nhlbi.nih.gov/lab/suffredini/proteomics/.

Twenty-four unvaccinated and ten vaccinated SARS-CoV-2 patients had plasma samples obtained at a median of 284 and 281 days post-infection, respectively, and were compared to HC (**Table 1**). Nineteen of 24 patients in the unvaccinated group were fully vaccinated after study enrollment but prior to the ten months blood sample collection. The median time from the last vaccine to blood sample collection was comparable in both groups (133 days in unvaccinated and 140 days in vaccinated patients) (**Table 1**). At ten months post-infection, unvaccinated and vaccinated SARS-CoV-2 patients clustered more closely together but continued to cluster separately from HC (**Figure 5A**). After adjusting for age, sex, and race, 858 unique proteins were differentially expressed in unvaccinated and vaccinated SARS-CoV-2 patients compared with HC (**Figures 5B-D**).

At ten months post-infection, unvaccinated patients had 53 overexpressed proteins involved in humoral immune response, innate immune response, immune effector process, and inflammatory response (C3b, CCL15, IL17RE, KRT1, DEFA5, TREM1), while vaccinated patients had 25 overexpressed proteins involved in cellular response to chemical stress (PSMA5), peroxisomal protein import, and folate metabolism compared with HC (**Figure 5E**). The graphical summary using IPA only showed enrichment for unvaccinated patients (**Supplementary Figure S6**).

At ten months post-infection, unvaccinated patients had 768 underexpressed proteins, whereas vaccinated patients had 634 underexpressed proteins compared with HC (**Figure 5F**). While both groups shared several pathways, including *VEGF-VEGFR2* signaling, intracellular protein transport, endocytosis, and actin filament-based processes, the number of proteins in each pathway differed across groups. For example, unvaccinated patients had 71 DEP in the *VEGF-VEGFR2* signaling pathway, while vaccinated patients had 60 DEP compared to HC. These observations were supported by IPA findings (**Supplementary Figure S7**).

When comparing expression of 1500 proteins across all time points, many overexpressed proteins during acute SARS-CoV-2 infection normalize by ten months post-infection, while many underexpressed proteins remained so at ten months (**Figure 5G**).

### Direct comparison of unvaccinated and vaccinated SARS-CoV-2 patients’ proteome reveals major differences in proteins involved in inflammatory and immune responses

To further explore differences that may be attributed to the vaccination status, we performed a direct comparison of unvaccinated and vaccinated patients after adjusting for age, sex, race, and days from symptom onset in the acute phase. Overall, unvaccinated patients exhibited overexpression of inflammatory, immune, and viral infection response compared to vaccinated patients which supports the observations described above. Further detail on the differentially over- and under- expressed proteins during acute infection and at three- and ten months post-infection are represented in **Supplementary Information** (text description, page 7) and **Supplementary Figures S8-S12**.

### Unsupervised hierarchical clustering identifies additional clinical factors, in addition to vaccination, contribute to different proteomic signatures after SARS-CoV-2 infection

**Figure 6:**
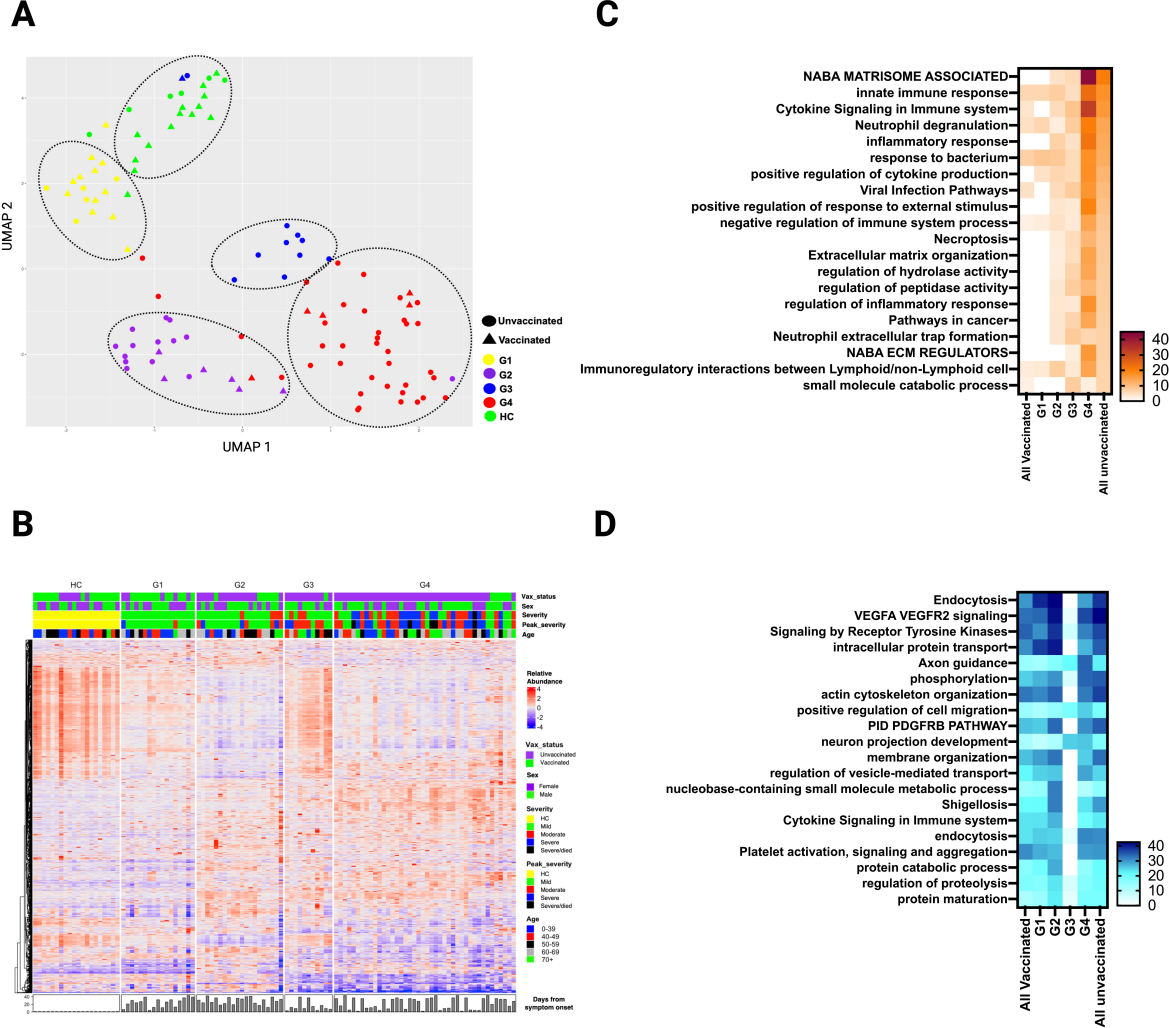
Plasma DEP in four clusters of SARS-CoV-2 patients identified by hierarchical clustering during the acute phase of infection. **(A)** UMAP of the plasma proteome from hierarchical clustering of 90 SARS-CoV-2 patients and 20 healthy controls in the acute phase of infection (circles - unvaccinated patients, triangles - vaccinated patients, and different colors denote the 4 SARS-CoV-2 groups and healthy controls. **(B)** Heatmap showing DEP among four SARS-CoV-2 clusters) and healthy controls. Each row represents a single protein; each column represents a patient. Pathway enrichment analysis of **(C)** overexpressed proteins and **(D)** underexpressed proteins in four SARS-CoV-2 clusters compared to all vaccinated and all unvaccinated from prior supervised clustering. An on-line summary of each protein across the different conditions analyzed is available at https://dir.nhlbi.nih.gov/lab/suffredini/proteomics/.

While comparison based on vaccination status yielded notable differences between unvaccinated and vaccinated SARS-CoV-2 patients, the groups did not fully separate (**Figure 1A**). Therefore, we applied a hierarchical clustering of the DEP, based on differential protein expression only, during acute illness that resulted in four distinct groups (G1 to G4) with variable distribution of clinical factors including vaccination status, comorbidities, severity of illness, time of sampling, viral variant, and others (**Figure 6A**; **Table 2**). For example, the vaccination rate for groups 1 to 4 were 71%, 30%, 9%, and 12% respectively. A heatmap of the 20 top pathways from the over- and under- expressed proteins among the four groups is shown in **Figures 6C, D**. Pathway analysis of the DEP showed that G1 resembled all vaccinated patients, while G2-G4 were more similar unvaccinated patients, with some variability in DEP contributions to pathway abnormalities across these groups (**Figure 6C**). Differentially overexpressed proteins involved in three top pathways among the four groups (G1-G4) and among unvaccinated and vaccinated SARS-CoV-2 patients during acute illness are summarized in **Supplementary Table S1**. G4 had the most abnormal profile with proteins involved in viral replication, inflammation, antiviral response, immune modulation, tissue damage response, and angiogenesis. Differentially underexpressed proteins, involved in three top pathways among the four groups (G1-G4) and among unvaccinated and vaccinated SARS-CoV-2 patients during acute illness are summarized in **Supplementary Table S2**. After adjusting for age, sex, race, and time from symptom onset, observed differences between G4 and G1-G3 remained (**Supplementary Table S3**). Analysis of the DEP from 32 patients at three months post-infection demonstrated differences across groups (**Supplementary Figures S13, S14**). Baseline characteristics of these 32 patients can be found in **Supplementary Table S4**).

**Table 2:** Characteristics of SARS-CoV-2 patients by proteomic group.

Characteristic	Group 1 (n=17)	Group 2 (n=20)	Group 3 (n=11)	Group 4 (n=42)
**Age, years**	50 (39-65)	54 (44-66)	57 (44-70)	60 (45-68)
**Sex (Female)**	8 (47%)	9 (45%)	4 (36%)	17 (40%)
Race-Ethnicity				
White Black Asian Latino	9 (52.9%) 6 (35.3%) - 2 (11.8%)	7 (35%) 5 (25%) 4 (20%) 4 (20%)	3 (27.3%) 5 (45.5%) 1 (9.1%) 2 (18.2%)	6 (14.3%) 27 (64.3%) - 9 (21.4%)
**Vaccination status**	12 (71%)	6 (30%)	1 (9%)	5 (12%) ^a^
Vaccine, n (%)				
BNT162b2 mRNA-1273 Ad26.COV2.S	10 (59%) 1 (6%) 1 (6%)	1 (5%) 4 (20%) 1 (5%)	- - 1 (9%)	2 (5%) 3 (7%) -
**Charlson Comorbidity Score**	1 (0-3.8)	1 (0-3.5)	1 (0-3)	2 (1-4)
**Time (days) from symptom onset**	31 (10-44)	35 (20-64)	15 (10-21)	9 (6-12)
NIAID Highest Ordinal scale				
4 (no oxygen) 5 (low-flow oxygen 6 (high-flow oxygen) 7 (MV/death)	15 (88%) 1 (6%) 1 (6%) -	13 (65%) 4 (20%) 3 (15%) -	3 (27%) 6 (55%) 2 (18%) -	8 (19%) 14 (33%) 15 (36%) 5 (12%)
NIAID Ordinal scale at time of sample				
4 (no oxygen) 5 (low-flow oxygen 6 (high-flow oxygen) 7 (MV/death)	17 (100%) - - -	8 (73%) 3 (27%) - -	16 (80%) 4 (20%) - -	12 (29%) 15 (36%) 14 (33%) 1 (2%)
SARS-CoV-2 variants				
Wuhan Alpha Delta Gamma Mu Omicron BA.1 other	6 (35%) - 10 (59%) 1 (6%) - - -	3 (15%) 4 (20%) 7 (35%) 5 (25%) - 1 (5%) -	1 (9%) 4 (36%) 2 (18%) 4 (36%) - - -	12 (29%) 6 (14%) 15 (36%) 5 (12%) 2 (5%) - 2 (5%)
**Any SARS-CoV-2 treatment**	6 (35%)	8 (40%)	9 (82%)	35 (83%)
**Any SARS-CoV-2 treatment (within 1 week of sample collection)**	2 (12%)	4 (20%)	8 (73%)	35 (83%)
**Any Steroids**	2 (12%)	6 (30%)	7 (64%)	31 (74%)
**Steroids within 1 week of sample collection**	1 (6%)	3 (15%)	7 (64%)	31 (74%)

Data are summarized by median (interquartile range) or n(%).

a 2 of the 5 vaccinated patients in G4 had moderate to severe end-stage renal disease and 1 had received a kidney transplant.

### Proteins associated with persistent chest radiological abnormalities at ten months post-infection

**Figure 7:**
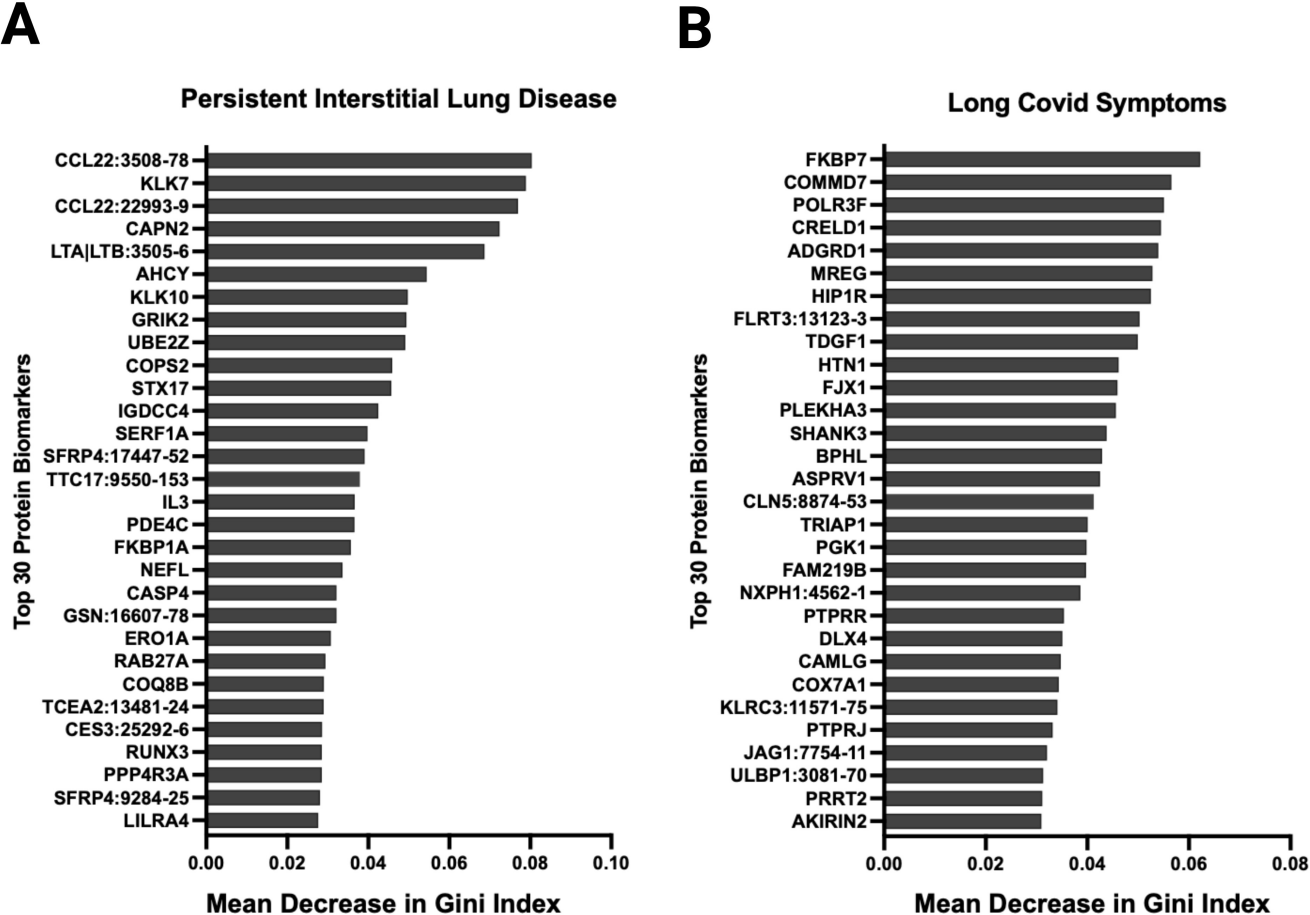
Random forest analysis at ten months post-infection identified several relevant proteins associated with relevant outcomes. Variable importance was measured using the mean decrease in Gini index. Variables with larger mean decreases in Gini index had greater variable importance for **(A)** persistent radiological abnormalities in SARS-CoV-2 patients at ten months post-infection. **(B)** any long SARS-CoV-2 symptoms ten months post-infection.

During acute SARS-CoV-2 infection, 12 of 31 had abnormal high resolution chest tomography (HRCT) findings. On follow-up imaging at ten months post-infection, nine of these 12 patients had persistent CT abnormalities consistent with interstitial lung disease. Among these nine patients, the mean percent predicted total lung capacity was 73.6% (SD 11.5) at three months post-infection which improved to 76% (SD 12.8) at ten months post-infection. Among these nine patients, the mean percent predicted diffusing capacity was 52.8% (SD 10.9) at three months which improved to 67.1% (SD 12.8) at ten months post-infection. Random forest analysis showed that the underexpression of macrophage-derived chemokine CCL22, which has been implicated in interstitial lung disease pathogenesis (22), had the highest variable importance that was associated with persistent CT abnormalities in our cohort (**Figure 7A**). Other proteins with high variable importance included KLK7, CAPN2, LTA|LTB, ACHY, and KLK10.

### Several proteins were associated with post-acute sequelae of SARS-CoV-2 at ten months post-infection

Thirty-six patients had symptoms data reported at ten months post-infection. Eighteen patients met clinical criteria for post-acute sequelae of SARS-CoV-2 (PASC), 8 with cardiovascular symptoms, 9 with musculoskeletal symptoms, and 15 with neurological symptoms. Random forest showed that FKBP7, COMMD7, POLR3F, CRELD1, and AGRD1 had the highest variable importance that was associated with PASC symptoms (**Figure 7B**). NOG, FAM17A1A, COL18A1, CDH6, and CHIA were associated with cardiopulmonary symptoms; LAS2, SPINT2, ALDH5A1, NOG, and FKBP14 with musculoskeletal symptoms; and NXPH1, CTSC, NCR3, HIP1R, and SCUB3 with neurologic symptoms (**Supplementary Figure S15**). In an exploratory fashion, we selected the most biologically relevant proteins, among the top 30 for each category, to build up a predictive model of symptoms at ten months (**Supplementary Figure S16**).

### Analysis of proteomic trajectories in SARS-CoV-2 patients during acute infection, and at three and ten months post-infection

**Figure 8:**
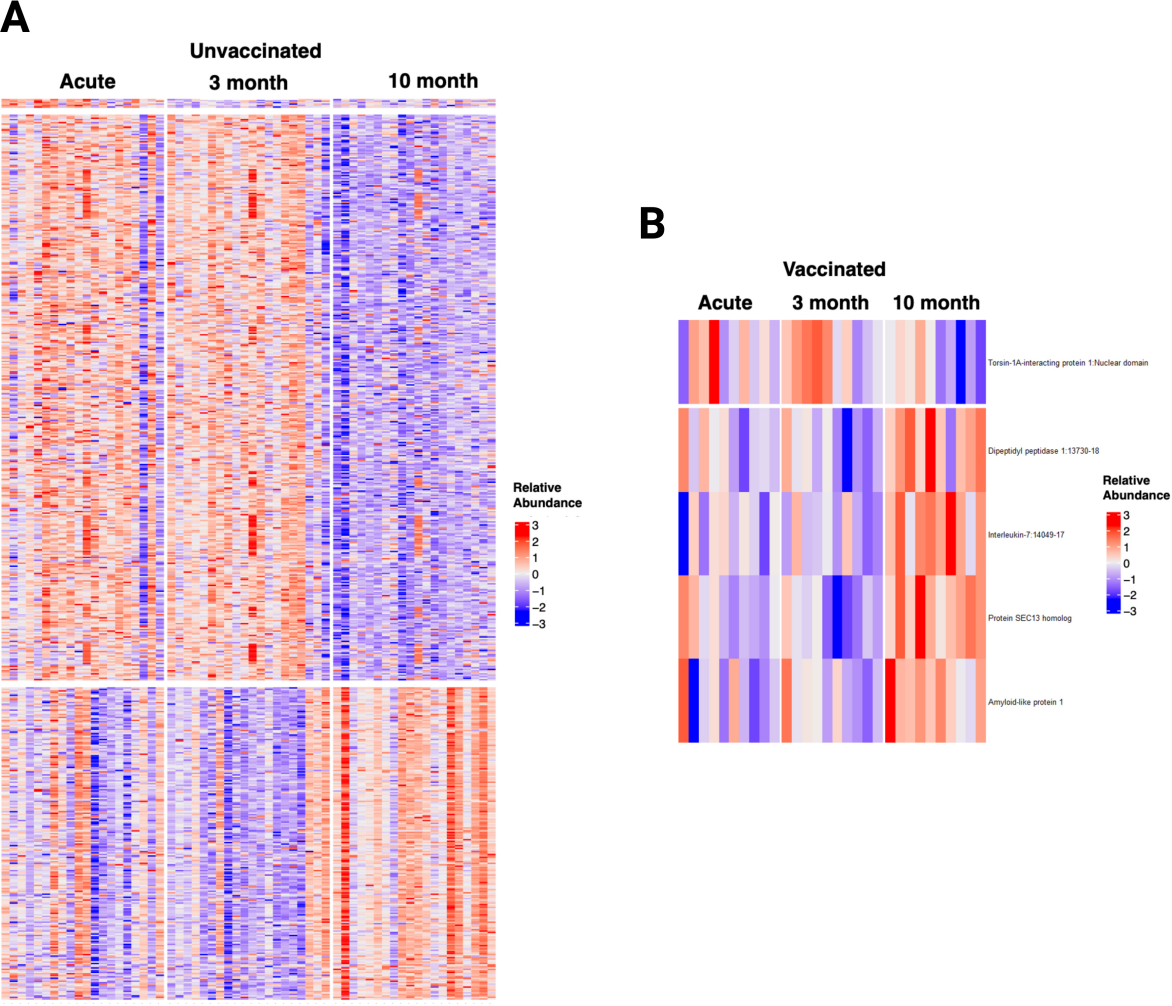
Longitudinal Analysis of the plasma proteome of SARS-CoV-2 patients identified relevant trends over time, particularly in unvaccinated patients. **(A)** Heatmap displaying the 602 proteins that changed significantly over time in unvaccinated SARS-CoV-2 patients. **(B)** Heatmap displaying the five proteins that changed significantly over time in vaccinated SARS-CoV-2 patients. Each row represents a single protein; each column represents a patient. The same patient is represented at each time point.

The time trend of the plasma proteome using mixed model analysis on the 1500 proteins in patients with repeated samples demonstrated that unvaccinated patients had 602 biomarkers that significantly changed over time (**Figure 8**). These proteins were clustered in 3 groups (**Supplementary Figure S17**): 6 proteins that decreased from acute to three months follow-up, 384 proteins that decreased from three months follow-up to ten months follow-up, and 212 proteins that increased from three months follow-up to ten months follow-up. Cluster 1 included proteins involved in neutrophil degranulation and intracellular chemical homeostasis. Cluster 2 included proteins involved in innate immune response, cytokine signaling in immune system, positive regulation of apoptotic process, regulation of IL-17 production, inflammatory response, among others. Cluster 3 included proteins involved in kit-receptor signaling, EGF-EGFR signaling, B cell receptor signaling, endocytosis, and filament-based process, among others. In contrast, the analysis only identified five proteins that significantly changed over time in vaccinated patients: one protein decreased from three months follow-up to ten months follow-up, and four proteins increased from three months follow-up to ten months follow-up. Sensitivity analysis of all the 90 patients with one to three repeated samples in the models yielded similar results for vaccinated patients and identified a fourth cluster of proteins in unvaccinated patients that increased from acute to three months follow-up. These proteins were involved in innate immune response, negative regulation of lymphocyte differentiation, HIF-1 signaling, cytokine signaling, and inflammatory response.

### Plasma proteome in vaccinated Omicron patients with breakthrough SARS-CoV-2 infections did not differ from vaccinated pre-Omicron patients

Using the customized panel, we analyzed the plasma proteome in 40 vaccinated patients with mild SARS-CoV-2 breakthrough infections according to SARS-CoV-2 variant. Fourteen patients had been infected through early December 2021 (Pre-Omicron) and 26 after January 2022 (Omicron). No differences were identified between these two groups (**Supplementary Figure S18**; **Supplementary Table S5**). Enrichment pathway analysis was similar in both groups when compared to HC (**Supplementary Figure S19**).

## Discussion

We conducted an analysis of the plasma proteome of SARS-CoV-2 patients using clustering based on either vaccination status alone or unsupervised clustering in the acute and recovery phases at three and ten months. The stratification of patients based on vaccination status revealed notable differences in their plasma proteomic responses. Acutely, unvaccinated patients exhibited a pronounced innate immune response with activation of pathways involved in acute inflammation and cytokine signaling, while vaccinated patients displayed an adaptive immune response with activation of the humoral response pathway. These data show that vaccination attenuates the acute inflammatory response to the virus accompanied by recruitment of adaptive immune responses. In the post-infection phase, unvaccinated patients had heightened protein expression in pathways associated with immune responses and inflammatory processes which remain detectable three months after the initial infection. In vaccinated patients, only 11 proteins were overexpressed, and these returned to normal levels by three months after the infection. At ten months, a panel of 1500 proteins identified during the acute phase, showed persistent overexpression of proteins involved in inflammation (C3b, CCL15, IL17RE) and immune responses (DEFA5, TREM1) only in unvaccinated patients. These findings emphasize the continued effects of SARS-CoV-2 infection on plasma protein expression and pathway activation, highlighting the lingering host responses in unvaccinated individuals.

In both unvaccinated and vaccinated patients, there were numerous proteins involved in immunity, cell function, cell signaling, and metabolic processes that were underexpressed compared to HC. This suggests SARS-CoV-2 demonstrates immunosuppressive effects on the systemic proteome during the acute infection. A study of transcriptomics from SARS-CoV-2 patients who died described downregulation of immune cell activation (T cells), metabolic processes (i.e. fatty acids) in colon tissue, and neurobiological processes (i.e. axon guidance and neuro projection guidance) in lung tissue (23). Similarly, our proteomic analysis also shows extensive downregulation of these pathways in both vaccinated and unvaccinated patients. In the post-infection phase at three and ten months, there was persistent protein underexpression of pathways essential for cellular function, signaling, and homeostasis (AKT1, MAPK14, HSPB1). However, the differences in the number of underexpressed proteins between unvaccinated and vaccinated individuals highlights the influence of vaccination on reducing the degree of underexpression and its implications for long-term immune response and delayed recovery. A study of 20 hospitalized patients with SARS-CoV-2 showed multiple systemic abnormalities in the proteome including upregulation of neutrophil degranulation, innate immune system, cytokine signaling in immune system, signaling by RHO GTPases, post translational protein modification, signaling by interleukins, adaptive immune system, among other pathways (14). Further, there was downregulation of several pathways including the innate immune system, developmental biology, post-translational protein modification, hemostasis, and extracellular matrix organization. Our study confirms these observations in a less severely ill cohort and extends the characterization of the systemic plasma proteome in SARS-CoV-2 patients interrogating a larger number of proteins over the course of ten months.

The clustering of our cohort based on vaccination status alone did not provide complete separation of vaccinated and unvaccinated patients. A recent epidemiologic study suggested that vaccination alone only partially reduces the risk of morbidity and mortality from SARS-CoV-2 infection (16). Unsupervised hierarchical clustering identified four SARS-CoV-2 groups with distinct proteomic profiles characterized by varying levels of immune activation, metabolic changes, and alterations in cellular processes. While the proteomic profile for G1 (72% vaccinated) was closer to vaccinated patients and G2, G3, and G4 (30, 9, and 12% vaccination rates respectively) closer to unvaccinated patients, the distribution of other clinical factors varied among the groups. This demonstrates the influence of additional clinical factors on the plasma proteome including age, sex, demographics, time of sampling, disease severity, and comorbidities. Examples include the similarities of the vaccinated group with G1 (72% vaccinated) both showing upregulation of annexin A3 (ANXA3), associated with pathogen clearance (24), and proteasome 20S subunit alpha 5 (PSMA5), associated with proteasome degradation of cellular proteins (25). In contrast, G2, G3, and G4 had persistent overexpression of proteins associated with inflammatory (e.g., IL18) and immune responses at three months follow-up. While G2, G3 and G4 shared several pathways, the number and the specific proteins involved in each pathway varied across the groups.

Studies of recovery of the plasma proteome after SARS-CoV-2 infection are influenced by several clinical and methodologic factors (e.g., targeted, or bottom-up proteomic analysis). Reports describe analyses of non-hospitalized patients that range from normalization of the proteome in the majority of patients after two weeks (6) or continued upregulation of proteins at five or six weeks post-infection (7, 8). Patients with moderate or severe disease have been found to have persistent changes in the plasma proteome at six months with changes in proteins associated with the extracellular matrix, immunological and inflammatory responses, platelet degranulation, and hemostasis pathways (5) and downregulation of proteins involved in wound healing, regulation of cell adhesion, and platelet activation (5, 26). In our study, we identified persistent proteome changes at ten-months post-infection in a cohort of patients with predominantly mild to moderate infection. In unvaccinated patients, 53 overexpressed proteins are involved in immune and inflammatory pathways. Their contribution to these processes depends in part on their temporal expression. Some proteins may have dual roles participating in both protective and potentially harmful pathways. For example, C3b is a key component of the complement system, which plays a dual role in enhancing immune defense against pathogens and potentially contributing to inflammatory damage. Similarly, IL17RE and TREM1 are crucial for initial defense, but they are also involved in deleterious inflammatory responses. While our study does not directly address whether these proteins are detrimental or protective in the context of reinfection, the chronic overexpression of inflammatory markers could contribute to exaggerated immune responses during subsequent infections or have implications for long-term health, such as the development of autoimmune conditions or chronic inflammatory diseases (27–29). These changes may be relevant to post-acute sequelae of SARS-CoV-2 and other viral infections associated with post-acute infection syndromes such Ebola, Dengue, H1N1, Epstein Barr, among others (17).

For this reason, we used a random forest machine learning approach to analyze the data on a subset of patients who had persistent symptoms and associated chest imaging. Several proteins including CCL22 showed to be associated with persistent radiological abnormalities at ten months post-infection. CCL22 is decreased in SARS-CoV-2 (30, 31) and may have a role in immune regulation (32). Conversely, CCL22 has also been reported to be elevated in bleomycin-induced pulmonary fibrosis (33) and in idiopathic pulmonary fibrosis (22). Proteins associated with any persistent symptom attributed to long COVID at ten months post-infection included FKBP7, which accelerates the folding of proteins during protein synthesis (34), and COMMD7, which associates with the NF-kappa-B complex and suppresses its transcriptional activity (34). Notably, NXPH1, which may play a role in the modulation of synaptic transmission (35), was the top protein associated with neurological symptoms. Larger validation studies are warranted to confirm these results.

Our study has several limitations such as enrollment timing variability and the impact of SARS-CoV-2 variants on the plasma proteome. While the enrollment timing varied, additional analyses, including the direct comparison between vaccinated and unvaccinated patients were adjusted for time from symptom onset. In unsupervised clustering, we found no signs of clusters based on SARS-COV-2 variants (**Table 1**). Further, analysis of the proteome of Omicron patients did not differ from pre-Omicron vaccinated patients.

## Conclusion

We describe the acute and long-term effects of SARS-CoV-2 vaccination on the plasma proteome. Our analysis shows that the dichotomy of vaccination versus no vaccination does not account for the additional clinical factors (e.g. comorbidities) that influence the resultant recovery of plasma proteome. The persistence of proteomic alterations in unvaccinated SARS-CoV-2 patients raises important questions about long-term altered immune responses and potential implications for later sequelae of SARS-CoV-2 infection, which may also be relevant in other viral infections associated with post-acute infection syndromes.

## Data Availability

The original contributions presented in the study are publicly available. This data can be found here: https://figshare.com/s/06c25f32b7ce9552e113?file=47998066.

## COVID-ARC study group collaborators

List of the collaborators of the COVID-Acute, Recovery, Convalescent (ARC) Study Group who contributed to the present investigation: Gloria Pastor^1,2^, Doris Swaim^1,3^, Julia Purdy^4^, Cheryl Chairez^4^, Mary McClaughlin^4^, Nicola Dee^4^, Kara A. Curl^4^, Rocco Caldararo^4^, Catherine Rehm^4^, Ulisses Santamaria^4^, Michelle DeVille^5^, Theresa Moriarity^5^, Diane Boyom Pouomogne^5^, Melissa Gonzales^5^, Aarthi Shenoy^5^

Affiliation of collaborators:

^1^Critical Care Medicine Department, Clinical Center, National Institutes of Health, Bethesda, Maryland, United States.

^2^Critical Care Medicine Branch, National Heart, Lung, and Blood, Institute, National Institutes of Health, Bethesda, MD, United States

^3^Applied/Developmental Research Directorate, Frederick National Laboratory for Cancer Research, Leidos Biomedical Research, Inc. Frederick, Maryland, United States.

^4^National Institute of Allergy and Infectious Diseases, Bethesda, Maryland, United States.

^5^MedStar Health Research Institute at Washington Hospital Center, Washington, D.C., United States
