# Supplementary material for: Alterations in the plasma proteome persist ten months after recovery from mild to moderate SARS-CoV-2 infection
Source: Front Immunol. 2024 Sep 11;15:1448780. doi: 10.3389/fimmu.2024.1448780 (PMC11422241; doi:10.3389/fimmu.2024.1448780)
Supplement: Supplementary file 1 [file DataSheet1.docx]

**Supplementary information**

**Table of contents**

**Text description**

-Specific variants (page 2)

-SOMAScan technical information (page 3)

-List of symptoms assessed at each visit (page 4)

-Ingenuity Pathway Analysis in SARS-CoV-2 in the acute phase of the infection (page 5)

-Ingenuity Pathway Analysis in SARS-CoV-2 patients three months post infection (page 6)

-Direct comparison between vaccinated and unvaccinated SARS-CoV-2 (page 7)

**Tables**

-Table S1. Differentially overexpressed proteins involved in three top pathways among the four groups (G1-G4), vaccinated, and unvaccinated SARS-CoV-2 individuals in the acute phase (page 8)

- Table S2. Differentially underexpressed proteins involved in three top pathways among the four groups (G1-G4), vaccinated, and unvaccinated SARS-CoV-2 individuals in the acute phase (page 9-11)

-Table S3. Differentially expressed proteins involved in three top pathways among the four groups (G1-G4), vaccinated, and unvaccinated SARS-CoV-2 individuals in the acute phase (page 12)

-Table S4. Baseline characteristics of the 32 SARS-CoV-2 patients among G1-G4 with follow-up at 3 months post-infection (page 13)

-Table S5. Differentially expressed proteins in vaccinated Omicron patients versus healthy controls and pre-Omicron patients in the acute and convalescent phases (page 14)

**Figures**

-Supplementary Figure S1 (page 15)

-Supplementary Figure S2 (page 16)

-Supplementary Figure S3 (page 17)

-Supplementary Figure S4 (page 18)

-Supplementary Figure S5 (page 29)

-Supplementary Figure S6 (page 20)

-Supplementary Figure S7 (page 21)

-Supplementary Figure S8 (page 22)

-Supplementary Figure S9 (page 23)

-Supplementary Figure S10 (page 24)

-Supplementary Figure S11 (page 25)

-Supplementary Figure S12 (page 26)

-Supplementary Figure S13 (page 27)

-Supplementary Figure S14 (page 28)

-Supplementary Figure S15 (page 29)

-Supplementary Figure S16 (page 30)

-Supplementary Figure S17 (page 31)

-Supplementary Figure S18 (page 32)

-Supplementary Figure S19 (page 33)

**Specific Variants**

The study utilized the Luciferase Immune-Complex Capture Assay (LICCA) to measure antibodies targeting receptor binding domains of viral variants. A plasmid containing synthetic SARS-CoV-2 Spike genes fused with luciferase and ferritin was transfected into 293 FT cells. After 48 hours, cell lysates were harvested, and Recombinant-Spike fusion protein antigens were incubated with patient serum samples. Antigen-antibody complexes were formed and captured on a protein-coated microplate, and luminescence was measured using a luciferase assay. SARS-CoV-2 variant references were provided, and results were expressed as fold changes in luminescence units compared to healthy controls, with a positive discrimination cutoff set at a fold change of >2. Further details can be found in previous publication^1^.

**SOMAScan technical information**

The SomaScan v4.0 and v4.1 Assays utilize 96 well plates, allocating eleven wells for control samples to manage batch effects and assess assay accuracy, precision, and buffer background over time. Each plate includes five pooled calibrator replicates, three pooled quality control (QC) replicates, and three buffer replicates. Twelve hybridization control SOMAmer® reagents are added for readout variability control. Control samples are repeatedly run during assay qualification, generating robust point estimates stored as references for each SOMAmer reagent's result. Plate calibration involves calculating the ratio of calibrator reference RFU value to plate-specific calibrator replicate median RFU value for each SOMAmer reagent. Adaptive normalization by maximum likelihood (ANML) is used for QC replicates and samples normalization. Post-calibration accuracy is estimated using the QC reference RFU value ratio. Plate-specific acceptance criteria include a scale factor between 0.4-2.5 and 85% of QC ratios between 0.8 and 1.2. Additional metrics such as calibration scale factor, QC sample precision, running precision, and buffer background are monitored daily for failures or trends. Triplicate QC samples since the SomaScan v4.0 Assay launch in April 2018 show production stability, with Coefficients of Variation (CVs) below 6% for half of the reagents and below 12% for 90% of the reagents using >3000 replicates in EDTA-plasma (median CV = 6%, 90th percentile CV = 12%). For full details, refer to the website (<https://somalogic.com/wp-content/uploads/2023/03/SomaScan-Assay-v4.1-Technical-Note.pdf>).

**List of symptoms assessed at each visit**

The in-person visits were performed by board certified physicians and included a detailed questionnaire at each visit which was further used for the telephone follow-ups. These symptoms included: Headache, fatigue, muscle aches, joint pain, back pain, vision, smell, taste, sleep, concentrating/memory, depression, anxiety, cough, shortness of breath, chest pain/ pressure, palpitations, limitations on walking, appetite, diarrhea, others.

**Ingenuity Pathway Analysis in SARS-CoV-2 patients in the Acute Phase of the infection**

For overexpressed proteins in the acute phase, the top canonical pathways in unvaccinated patients included *pathogen induced cytokine storm signaling*, *airway pathology in chronic obstructive pulmonary diseases*, *the role of hypercytokinemia/hyperchemokinemia in the pathogenesis of influenza*, *multiple sclerosis signaling*, and *RXR/RXR activation*. Among other relevant pathways, unvaccinated patients had enrichment of several cytokine signaling pathways including *IL33 signaling* (CREBBP, CTSG, ELANE, H2AZ1, H2BC12, H2BC26, ICAM3, IL5, IL18, IL17F, IL1RN, MAPK13, MMP9, and TPSG1) and *acute phase response signaling* (C3, CRP, F8, HAMP, IL18, IL1RN, MAPK-13, SAA1, SAA2, SERPINA3, SERPING1, vWF), *neutrophil extracellular trap signaling pathway* (CLEC7A, CTSG, ELANE, FCAR, FCGR3A/FCGR3B, HIF1A, IFNA6, IFNA16, ITGB2, MAPK13, MMP9, MPO, NDUFB11, PLAAT2, PRTN3, PYCARD), among others.

In contrast, the top canonical pathways in vaccinated patients included *S-adenosyl-L-methionine biosynthesis*, *inhibition of ARE-Mediated mRNA degradation pathway*, *FAT10 signaling pathway*, *granzyme B signaling*, and *methionine degradation I*. Of note, other important pathways included the humoral immune response (B2M and PSMB6 in the *antigen presentation pathway*), intracellular and second messenger signaling (AGO2, PSMA5, PSMB6, PSMD9, YWHAE in the *inhibition of ARE-mediated mRNA degradation pathway*) and cellular immune response (MPO, TREM1 in the *TREM1 signaling pathway*).

For underexpression, The top canonical pathways in both vaccinated and unvaccinated patients included *acute phase, integrin signaling, insulin receptor signaling, hepatic fibrosis signaling pathway, VEGF signaling,* and *FC Epsilon RI signaling.*

**Ingenuity Pathway Analysis in SARS-CoV-2 patients three months post infection**

For overexpressed proteins three months post infection, the top canonical pathways in unvaccinated patients included *multiple sclerosis signaling pathway, osteoarthritis pathway, macrophage classical activation signaling pathway, RAR activation,* and *erythropoietin signaling pathway*. In vaccinated patients, the top canonical pathways included *apelin adipocytes signaling pathway, UDP-N-acetyl-D-glucosamine biosynthesis II, superoxide radicals degradation,* and *ethanol degradation IV*.

For underexpression three months post infection, both groups had enrichment of important pathways including *integrin signaling, actin cytoskeleton signaling, Fcy receptor-mediated phagocytosis in macrophages and monocytes,* and *ERK/MAPK signaling.*

**Direct comparison between vaccinated and unvaccinated SARS-CoV-2 patients demonstrates major differences in their systemic proteome**

Compared to vaccinated patients, our results demonstrate the presence of 347 *overexpressed* and 338 *underexpressed* proteins in unvaccinated patients in the acute phase. Supplemental Fig. S4a. displays the heatmap using 1981 differentially expressed proteins between unvaccinated and vaccinated patients. HC are included for reference only. Supplemental Fig. S4c displays the volcano plot of the differentially overexpressed and underexpressed proteins in the acute phase between unvaccinated and vaccinated patients.

Pathway analysis of the overexpressed proteins showed that unvaccinated patients had enrichment of pathways involved in *cytokine signaling in immune system, viral infection,* and *defense response to gram-positive bacterium,* among other pathways (Supplemental Fig. S5). Pathway analysis of underexpressed proteins showed enrichment of *matrisome associated, axon guidance, and cell-cell adhesion, among other* pathways (Supplemental Fig. S6). On follow-up three months post-infection, we observed 89 *overexpressed* and 116 *underexpressed* proteins in unvaccinated patients compared to vaccinated (Supplemental Fig. S5b and S5d). Enrichment pathway analysis of these overexpressed and underexpressed proteins in unvaccinated patients can be found in Supplemental Fig. S7-S8. No differences were seen between vaccinated and unvaccinated patients using the customized panel analyzed in the ten month post infection phase.

**Tables**

**e-Table 1. Differentially overexpressed proteins involved in 3 top pathways among the four groups (G1-G4), vaccinated, and unvaccinated SARS-CoV-2 individuals in the acute phase**

| **Pathway** | **Groups** | **N** | **Proteins** |
| --- | --- | --- | --- |
| **R-HSA-1474244: Extracellular matrix organization** | Vaccinated | 0 | **-** |
|  | G1 | 0 | - |
|  | G2 | 11 | ADAM10, COL6A3, IBSP, ITGB1, ITGB2, VCAM1, VTN, SDC3, OPTC, TMPRSS6, COL28A1 |
|  | G3 | 13 | FGG, FN1, IBSP, ICAM3, ITGB2, LAMA4, NID1, TIMP1, VTN, VWF, GDF5, NID2, TMPRSS6 |
|  | G4 | 37 | ADAM10, BSG, CAPN2, CMA1, CTSG, DCN, ELANE, IBSP, ICAM3, ITGAL, ITGB2, ITGB7, MATN3, MMP9, MMP17, NID1, FURIN, PPIB, HTRA1, TGFB1, TGFB3, TIMP1, VTN, VWF, ADAM12, GDF5, ADAM15, ADAMTS3, ADAMTS1, FBLN5, NID2, OPTC, BMP10, SPOCK3, TMPRSS6, SCUBE3, AGRN |
|  | Unvaccinated | 23 | ADAM10, CAPN2, CTSG, ELANE, IBSP, ICAM3, ITGB2, LAMA4, MATN3, MMP9, MMP17, FURIN, HTRA1, TIMP1, VTN, VWF, GDF5, CASK, ADAMTS3, FBLN5, OPTC, TMPRSS6, AGRN |
|  | | | |
| **GO 0006954: inflammatory response** | Vaccinated | 0 | - |
|  | G1 | 0 | - |
|  | G2 | 26 | C3, DHX9, FCER1A, FCGR3B, HIF1A, HK1, IL5, ITGB1, ITGB2, LGALS9, S100A8, S100A9, TFR2, TLR1, TNF, TNFSF4, VCAM1, GPR68, IL18R1, PSTPIP1, TXNIP, IL17C, FOXP3, IL17F, IL17RE, TAC4 |
|  | G3 | 18 | SERPINA3, C3, F8, FCGR3B, FN1, HIF1A, IFI16, IL5, IL18, ITGB2, LGALS9, S100A8, S100A9, TIMP1, TLR1, GPR68, PYCARD, CLEC7A |
|  | G4 | 64 | SERPINA3, ALOX5, BPGM, C3, CRP, CSF1, ELANE, F8, FCGR1A, FCGR3B, B4GALT1, GRN, HIF1A, HMGB2, IFI16, IFNGR2, IL1B, IL1RN, IL5, IL6, CXCL8, IL18, CXCL10, ITGAL, ITGB2, LBP, LGALS9, MDK, OLR1, SERPINA1, PLA2G2A, PRKCZ, PTN, PTX3, S100A8, S100A9, S100A12, SAA1, SAA2, CCL7, CCL13, CCL15, CCL23, CXCL11, TGFB1, TIMP1, TLR1, GPR68, PLA2G10, IL18R1, PSTPIP1, IL1RL1, CHST2, CCL26, APOL2, IL36B, PYCARD, IL17D, HAMP, PROK2, CLEC7A, HAVCR2, IL17F, NLRP4 |
|  | Unvaccinated | 42 | SERPINA3, BPGM, C3, CRP, CSF1, ELANE, F3, F8, FCGR3B, GRN, HIF1A, HMGB2, IFI16, IL1RN, IL5, IL18, CXCL10, ITGB2, LGALS9, MDK, CXCL9, OLR1, S100A8, S100A9, S100A12, SAA1, SAA2, CCL7, CCL15, TIMP1, TLR1, GPR68, IL18R1, PSTPIP1, CCL26, PYCARD, IL17D, HAMP, PROK2, CLEC7A, IL17F, NLRP4 |
|  | | | |
| **R-HSA-1280215: Cytokine Signaling in Immune system** | Vaccinated | 11 | B2M, CA1, ILF3, YBX1, PRTN3, PSMA5, PSMB6, PSMD9, FSCN1, UBB, ISG15 |
|  | G1 | 7 | B2M, CA1, ILF3, IRF4, PSMA5, UBB, ISG15 |
|  | G2 | 30 | BIRC3, CA1, DHX9, HIF1A, IFNA6, IFNA16, IL5, ILF3, IRF1, IRF2, ITGB1, ITGB2, JUN, LGALS9, PLCG1, PRTN3, PSMB6, SOX2, TNF, TNFSF4, VCAM1, PRKRA, IL18R1, ISG15, IL17C, DUS2, TRIM5, IL17F, IL17RE, GBP6 |
|  | G3 | 30 | ABL2, BIRC3, ATF1, B2M, TNFRSF8, EIF4A1, FN1, GBP1, HGF, HIF1A, HNRNPA2B1, IL5, IL15RA, IL18, ILF3, ITGB2, LGALS9, MX1, OAS1, PRTN3, PSMA2, SEC13, SOD1, STAT1, TIMP1, VEGFA, H3C1, PRKRA, ISG15, RIGI |
|  | G4 | 91 | ABL2, ALOX5, BIRC3, ATF1, B2M, BCL2, CA1, CD4, TNFRSF8, CSF1, CTSG, FCGR1A, FLT3LG, GBP1, GBP2, HGF, HIF1A, HLA-G, HNRNPA2B1, HSPA1A, HSPA8, IFNA6, IFNA10, IFNA16, IFNGR2, IL1B, IL1RN, IL5, IL6, CXCL8, IL12RB1, IL15RA, IL18, ILF3, CXCL10, IRF2, IRF4, ITGB2, LBP, LCP1, LGALS9, LIF, LMNB1, MCL1, MMP9, MX1, NPM1, YBX1, OAS1, PML, PRLR, PRTN3, PSMA1, PSMA2, PSMB4, PSMB6, PSME2, PTGS2, RPS27A, S100A12, SAA1, SEC13, SNRPA1, SOD1, SOX2, TGFB1, TIMP1, UBB, VEGFA, VIM, H3C1, CUL1, PRKRA, IL18R1, SOCS3, IL1RL1, GSTO1, ISG15, IRF9, TNFRSF13B, RIGI, IL36B, IL21R, IL22RA1, EDA2R, HAVCR2, TRIM5, TIFA, IL17F, IFNL2, IFNL1 |
|  | Unvaccinated | 56 | ABL2, BIRC3, ATF1, B2M, CA1, CD4, TNFRSF8, CSF1, CTSG, FLT3LG, GBP1, HGF, HIF1A, HNRNPA2B1, HSPA1A, IFNA6, IFNA16, IL1RN, IL5, IL12RB1, IL15RA, IL18, ILF3, CXCL10, IRF2, IRF4, ITGB2, LCP1, LGALS9, MMP9, MX1, NPM1, YBX1, OAS1, PRTN3, PSMA2, PSMB4, S100A12, SAA1, SNRPA1, SOX2, TIMP1, UBB, H3C1, PRKRA, IL18R1, GSTO1, ISG15, TNFRSF13B, RIGI, IL21R, IL22RA1, EDA2R, TRIM5, IL17F, IFNL1 |

**e-Table 2. Differentially underexpressed proteins involved in 3 top pathways among the four groups (G1-G4), vaccinated, and unvaccinated SARS-CoV-2 individuals in the acute phase**

| **Pathway** | **Groups** | **N** | **Proteins** |
| --- | --- | --- | --- |
| **WP3888: VEGFA VEGFR2 signaling** | Vaccinated | 69 | AKT1, ALDOA, ARF4, RHOA, RHOC, BCL2L1, CBL, CFL1, CLIC1, MAPK14, CSK, CSRP1, CTNNB1, DECR1, DOK1, EIF4G1, EIF4G2, ERN1, PTK2B, FLII, FYN, GRB2, GRB10, GSK3B, HSPB1, HSP90AA1, LDHA, MYO6, NAP1L1, PDPK1, PFN1, PGK1, PIK3CA, PLA2G4A, PPP3CA, PRKAA2, PRKCA, PRKCB, MAPK3, PTPN1, PTPN6, PTPN11, RAB4A, RAF1, SHC1, SRC, STAT3, STAT6, TFAM, VCL, EZR, MKNK1, NUMB, ADAM9, RAB11A, INPP4B, HGS, LRRFIP2, MAPKAPK2, ROCK2, PDIA6, GIPC1, FHOD1, TMOD3, EPN1, LRRC59, SH3BGRL3, EIF2A, AKT1S1 |
|  | G1 | 74 | AKT1, ALDOA, ARF4, RHOA, RHOC, BCL2L1, CBL, CFL1, CLIC1, MAPK14, CSK, CSRP1, CSRP2, CTNNB1, DECR1, DOK1, EIF4G1, EIF4G2, ERN1, PTK2B, FLII, FYN, GAB1, GRB2, GRB10, GSK3B, HSPB1, HSP90AA1, LDHA, MYO6, NAP1L1, PDPK1, PFN1, PIK3CA, PLA2G4A, PPP3CA, PRKAA2, PRKCA, PRKCB, MAPK1, MAPK3, PSMD11, PTPN1, PTPN6, PTPN11, RAB4A, RAB5A, RAF1, SHC1, SRC, SRPK1, STAT3, STAT6, TFAM, TKT, VCL, EZR, MKNK1, NUMB, RAB11A, INPP4B, HGS, LRRFIP2, ROCK2, PDIA6, CCT7, GIPC1, FHOD1, TMOD3, EPN1, LRRC59, SH3BGRL3, EIF2A, AKT1S1 |
|  | G2 | 92 | AKT1, ALDOA, ARF4, RHOA, RHOC, CBL, CDC42, CFL1, CLIC1, CNP, CRK, MAPK14, CSK, CSRP1, CTNNA1, DECR1, DOK1, EIF4E, EIF4G1, EIF4G2, ERN1, PTK2B, FLII, FYN, GAB1, GAPDH, GPX1, GRB2, GRB10, GSK3B, HSPB1, HSP90AA1, LDHA, MYO6, NAP1L1, NFKB1, PNP, PDPK1, PFN1, PGD, PGK1, PIK3CA, PLA2G4A, PPP3CA, PRKAA2, PRKCA, PRKCB, PRKCI, MAPK1, MAPK3, MAPK8, MAP2K1, PSMD11, PTPN1, PTPN6, PTPN11, RAB4A, RAB5A, RAC1, RAF1, SHC1, SRC, SRPK1, STAT1, STAT3, STAT6, TFAM, TKT, VCL, EZR, MKNK1, NUMB, ADAM9, RAB11A, NAPA, INPP4B, USP10, HGS, LRRFIP2, MAPKAPK2, ROCK2, PDIA6, CCT7, GIPC1, FHOD1, TMOD3, EPN1, LRRC59, SH3BGRL3, EIF2A, AKT1S1, AMOT |
|  | G3 | 11 | JAG1, CDH5, ENG, MMP2, PLAU, MAP2K4, TNXB, ADAM9, TMOD3, CHAC1, SEMA6D |
|  | G4 | 88 | AKT1, ALB, ARF4, RHOA, RHOC, BCL2L1, CBL, CDC42, CFL1, CLIC1, MAPK14, CSK, CSRP1, CSRP2, CTNNA1, CTNNB1, DECR1, DOK1, EIF4E, EIF4G1, EIF4G2, ENG, ERN1, PTK2B, FLII, FYN, GLUD1, GRB2, GRB10, GSK3B, HSPB1, IGFBP3, MYO6, NAP1L1, NFKB1, PDPK1, PFN1, PGK1, PIK3CA, PLA2G4A, PPP3CA, PRKAA2, PRKCA, PRKCB, MAPK1, MAPK3, MAPK8, MAP2K6, PTPN1, PTPN11, RAB4A, RAB5A, RAC1, RAF1, MAP2K4, SET, SHC1, SOD2, SRC, SRPK1, STAT3, STAT6, TFAM, TNXB, VCL, MKNK1, NUMB, ADAM9, RAB11A, INPP4B, HGS, LRRFIP2, MAPKAPK2, ROCK2, PDIA6, CCT7, GIPC1, FHOD1, TMOD3, EPN1, CYCS, LRRC59, MMRN2, SEMA6D, SH3BGRL3, EIF2A, AKT1S1, AMOT |
|  | Unvaccinated | 91 | AKT1, ALB, ARF4, RHOA, RHOC, CBL, CDC42, CFL1, CLIC1, MAPK14, CSK, CSRP1, CSRP2, CTNNA1, DECR1, DOK1, EIF4E, EIF4G1, EIF4G2, ENG, ERN1, PTK2B, FLII, FYN, GAB1, GAPDH, GLUD1, GPX1, GRB2, GRB10, GSK3B, IGFBP3, MYO6, NAP1L1, NFKB1, PDPK1, PFN1, PGD, PGK1, PIK3CA, PLA2G4A, PPP3CA, PRKAA2, PRKCA, PRKCB, PRKCI, MAPK1, MAPK3, MAPK8, MAP2K1, MAP2K3, PSMD11, PTPN1, PTPN6, PTPN11, RAB4A, RAB5A, RAC1, RAF1, MAP2K4, SET, SHC1, SRC, SRPK1, STAT3, STAT6, TFAM, VCL, EZR, MKNK1, NUMB, ADAM9, RAB11A, INPP4B, HGS, LRRFIP2, MAPKAPK2, ROCK2, PDIA6, CCT7, GIPC1, FHOD1, TMOD3, EPN1, CYCS, LRRC59, MMRN2, SH3BGRL3, EIF2A, AKT1S1, AMOT |
|  |  |  |  |
| **GO0030029: actin filament-based process** | Vaccinated | 83 | ACTN4, ACTN1, ADD2, AIF1, ALDOA, RHOA, RHOC, ARHGAP6, ARRB1, CALD1, CAMK2D, CAPZA1, CFL1, CNN2, CSRP1, DPYSL3, PTK2B, FER, FLII, FLNA, GMFB, GRB2, IKBKB, JAK2, MYL6, MYO6, OPHN1, PAWR, PDPK1, PFN1, PIK3CA, PKP2, PLEK, PTPN1, RAB13, RAC3, RAP1GDS1, SHC1, SNTA1, SRC, TPM4, VASP, VIL1, EZR, WAS, ZYX, CSRP3, TAGLN2, NCK2, PDLIM1, TRIP10, NHERF1, ROCK2, GMFG, ARHGAP25, HDAC6, ARPC1B, ARPC2, MYL9, CAP1, PDLIM5, DSTN, PACSIN2, TWF2, SYNE2, RHOQ, CD2AP, NAA80, DBNL, FHOD1, TMOD3, TMOD2, PACSIN1, LIMA1, GHRL, RHOT1, ENAH, MICAL1, PDCL3, LIMD2, SETD3, SPATC1L, PPP1R9B |
|  | G1 | 86 | ACTN4, ACTN1, ADD2, AIF1, ALDOA, RHOA, RHOC, ARHGAP6, ARRB1, CALD1, CAMK2D, CAPZA1, CFL1, CNN2, CSRP1, CSRP2, DIAPH1, DPYSL3, PTK2B, FER, FLII, FLNA, GMFB, GRB2, HCLS1, IKBKB, JAK2, MYO6, OPHN1, PAWR, PDPK1, PFN1, PIK3CA, PKP2, PLEK, PTPN1, RAB13, RAC3, RAP1GDS1, SHC1, SNTA1, SRC, TPM4, VASP, VIL1, EZR, WAS, ZYX, CSRP3, TAGLN2, NCK2, PDLIM1, TRIP10, NHERF1, ROCK2, GMFG, ARHGAP25, HDAC6, PDCD6IP, ARPC1B, ARPC2, MYL9, CAP1, PDLIM5, DSTN, PACSIN2, TWF2, SYNE2, RHOQ, ACAP2, CD2AP, NAA80, DBNL, FHOD1, TMOD3, TMOD2, PACSIN1, LIMA1, RHOT1, ENAH, ELMO2, MICAL1, PDCL3, LIMD2, SETD3, PPP1R9B |
|  | G2 | 101 | ACTN4, ACTN1, ADD2, AIF1, ALDOA, RHOA, RHOC, ARHGAP6, ARRB1, CALD1, CAMK2D, CAPZA1, CDC42, CFL1, CNN2, CRK, CSRP1, DIAPH1, DPYSL3, PTK2B, FER, FGF7, FLII, FLNA, GRB2, HCLS1, IKBKB, JAK2, MYL6, MYO6, OPHN1, PAWR, PDPK1, PFN1, PIK3CA, PKP2, PLEK, PRKCI, TWF1, PTPN1, RAB13, RAC1, RAC2, RAC3, RAN, RAP1GDS1, SHC1, SMTN, SNTA1, SRC, TPM4, VASP, VIL1, EZR, WAS, ZYX, CSRP3, TAGLN2, NCK2, CDC42BPA, PDLIM1, TRIP10, NHERF1, ROCK2, ARHGAP25, HDAC6, PDCD6IP, ARPC3, ARPC1B, ARPC2, MYL9, CAP1, PDLIM5, DSTN, PACSIN2, TWF2, SYNE2, SRGAP2, RHOQ, ACAP2, CD2AP, NAA80, VPS4A, DBNL, FHOD1, TMOD3, TMOD2, PACSIN1, LIMA1, GHRL, RHOT1, ENAH, PDXP, ELMO2, MICAL1, PDCL3, LIMD2, SETD3, PPP1R9B, AGAP2, AMOT |
|  | G4 | 97 | ACTN4, ACTN1, ADD2, AIF1, RHOA, RHOC, ARHGAP6, ARRB1, BRAF, CALD1, CAMK2D, CAPZA1, CDC42, CFL1, CNN1, CNN2, CSRP1, CSRP2, DPYSL3, DMTN, PTK2B, FER, FLII, FLNA, GRB2, GSN, IKBKB, JAK2, KIT, MYO6, OPHN1, PAWR, PDGFRB, PDPK1, PFN1, PIK3CA, PKP2, PLEK, PTPN1, RAB13, RAC1, RAC2, RAP1GDS1, SCN2B, SHC1, SMTN, SNTA1, SRC, TJP1, TNXB, TPM2, TPM4, VASP, VIL1, WAS, ZYX, TAGLN2, NCK2, PDLIM1, TRIP10, NHERF1, ROCK2, ARHGAP25, HDAC6, ARPC3, ARPC1B, ARPC2, MYL9, CAP1, PDLIM5, DSTN, PACSIN2, TWF2, ARHGAP26, CD2AP, ARFIP2, NAA80, VPS4A, DBNL, FHOD1, TMOD3, TMOD2, PACSIN1, F11R, LIMA1, GHRL, RHOT1, ENAH, MICAL1, PDCL3, LIMD2, ANTXR1, SETD3, SPATC1L, PPP1R9B, AGAP2, AMOT |
|  | Unvaxed | 101 | ACTN4, ACTN1, ADD2, AIF1, RHOA, RHOC, ARHGAP6, ARRB1, BRAF, CALD1, CAMK2D, CAPZA1, CDC42, CFL1, CNN1, CNN2, CSRP1, CSRP2, DPYSL3, PTK2B, FER, FLII, FLNA, GRB2, GSN, IKBKB, JAK2, KIT, MYL6, MYO6, OPHN1, PAWR, PDGFRB, PDPK1, PFN1, PIK3CA, PKP2, PLEK, PRKAR1A, PRKCI, PTPN1, RAB13, RAC1, RAC2, RAC3, RAP1GDS1, SHC1, SMTN, SNTA1, SRC, TPM4, VASP, VIL1, EZR, WAS, ZYX, CSRP3, TAGLN2, NCK2, PDLIM1, TRIP10, NHERF1, ROCK2, ARHGAP25, HDAC6, PDCD6IP, ARPC3, ARPC1B, ARPC2, MYL9, CAP1, PDLIM5, DSTN, PACSIN2, TWF2, SRGAP2, RHOQ, ACAP2, CD2AP, ARFIP2, NAA80, VPS4A, DBNL, FHOD1, TMOD3, TMOD2, PACSIN1, LIMA1, GHRL, RHOT1, ENAH, ELMO2, MICAL1, PDCL3, LIMD2, ANTXR1, SETD3, SPATC1L, PPP1R9B, AGAP2, AMOT |
|  |  |  |  |
| **hsa04144: Endocytosis** | Vaccinated | 51 | GRK2, AP2A2, ARF4, ARF5, RHOA, ARRB1, CAPZA1, CBL, DAB2, DNM1, DNM2, SMAD2, SMAD3, CHMP1A, RAB4A, RAB5C, SH3GL2, SH3GL3, SRC, TSG101, RAB7A, SNX4, RAB11A, USP8, HGS, RAB11B, CYTH3, VPS26A, RAB11FIP3, ARPC1B, ARPC2, RNF41, EHD1, RAB31, WWP1, SPART, CHMP2B, LDLRAP1, SNX5, EPN1, SNX12, EHD3, SPG21, CHMP3, ASAP3, EPS15L1, SMAP1, CHMP6, ARFGAP2, MVB12B, ZFYVE27 |
|  | G1 | 63 | GRK2, AP2A2, AP2B1, ARF4, RHOA, ARRB1, CAPZA1, CBL, DAB2, DNM1, DNM2, SMAD2, SMAD3, CHMP1A, RAB4A, RAB5A, RAB5C, SH3GL2, SH3GL3, SNX1, SRC, TSG101, RAB7A, SNX4, RAB11A, USP8, RABEP1, HGS, RAB11B, CYTH3, VPS4B, VPS26A, RAB11FIP3, IST1, PDCD6IP, ARPC1B, ARPC2, RNF41, STAMBP, EHD1, RAB31, WWP1, SPART, ACAP2, CHMP2B, LDLRAP1, SNX5, CHMP2A, EPN1, SNX12, EHD3, SPG21, VTA1, CHMP3, ASAP3, CHMP1B, EPS15L1, SMAP1, CHMP6, VPS25, ARFGAP2, MVB12B, ZFYVE27 |
|  | G2 | 73 | GRK2, AP2A2, AP2B1, ARF1, ARF4, RHOA, ARRB1, CAPZA1, CBL, CDC42, CLTA, DAB2, DNM1, DNM2, IL2RG, SMAD2, SMAD3, CHMP1A, PRKCI, RAB4A, RAB5A, RAB5B, RAB5C, SH3GL2, SH3GL3, SRC, TSG101, RAB7A, SNX4, RAB11A, USP8, RABEP1, HGS, RAB11B, CYTH3, VPS26A, RAB11FIP3, IST1, PDCD6IP, ARPC3, ARPC1B, ARPC2, RNF41, STAMBP, EHD1, RAB31, WWP1, SPART, ACAP2, CHMP2B, LDLRAP1, CYTH4, SNX5, VPS4A, CHMP2A, CHMP4A, EPN1, SNX12, EHD3, SPG21, VTA1, CHMP3, ASAP3, CHMP1B, RAB22A, EPS15L1, SMAP1, CHMP6, VPS25, ARFGAP2, MVB12B, AGAP2, ZFYVE27 |
|  | G4 | 60 | GRK2, AP2A2, AP2B1, ARF4, ARF5, RHOA, ARRB1, CAPZA1, CBL, CDC42, DAB2, DNM1, DNM2, IGF1R, SMAD2, SMAD3, CHMP1A, RAB4A, RAB5A, RAB5B, RAB5C, SH3GL2, SH3GL3, SRC, TSG101, RAB7A, SNX4, RAB11A, USP8, RABEP1, HGS, RAB11B, CYTH3, RAB11FIP3, IST1, ARPC3, ARPC1B, ARPC2, EHD1, RAB31, WWP1, SPART, CHMP2B, LDLRAP1, SNX5, VPS4A, EPN1, SNX12, EHD3, VPS28, SPG21, CHMP3, RAB22A, EPS15L1, SMAP1, CHMP6, PSD2, ARFGAP2, MVB12B, AGAP2 |
|  | Unvaccinated | 67 | GRK2, AP2A2, AP2B1, ARF4, ARF5, RHOA, ARRB1, CAPZA1, CBL, CDC42, DAB2, DNM1, DNM2, SMAD2, SMAD3, CHMP1A, PRKCI, RAB4A, RAB5A, RAB5B, RAB5C, SH3GL2, SH3GL3, SRC, TSG101, RAB7A, SNX4, RAB11A, USP8, RABEP1, HGS, RAB11B, CYTH3, VPS26A, RAB11FIP3, IST1, PDCD6IP, ARPC3, ARPC1B, ARPC2, STAMBP, EHD1, RAB31, WWP1, SPART, ACAP2, CHMP2B, LDLRAP1, SNX5, VPS4A, CHMP2A, EPN1, SNX12, EHD3, SPG21, VTA1, CHMP3, ASAP3, CHMP1B, RAB22A, EPS15L1, SMAP1, CHMP6, VPS25, ARFGAP2, MVB12B, AGAP2 |

**e-Table 3. Differentially expressed proteins involved in 3 top pathways among the four groups (G1-G4), vaccinated, and unvaccinated SARS-CoV-2 individuals in the acute phase**

| **Comparison*** | **Differentially Expressed proteins** | |
| --- | --- | --- |
|  | **Overexpressed** | **Underexpressed** |
| SARS-CoV-2 G4 vs G1 | 317 | 172 |
| SARS-CoV-2 G4 vs G2 | 349 | 94 |
| SARS-CoV-2 G4 vs G3 | 101 | 741 |

*analysis adjusted for age, sex race, and time from symptom onset

**e-Table 4. Baseline characteristics of the 32 SARS-CoV-2 patients among G1-G4 with follow-up at 3 months post-infection**

|  | **Group 1**  **(n=8)** | **Group 2**  **(n=11)** | **Group 3**  **(n=7)** | **Group 4**  **(n=6)** |
| --- | --- | --- | --- | --- |
| **Age, median (25^th^-75^th^ percentiles)** | 43.6 ± 11.8 | 47.5 ± 15.9 | 52.7 ±15 | 47.8 ±15.5 |
| **Sex (Female)** | 4 (50%) | 4 (36.4%) | 2 (28.6%) | 2 (33.3%) |
| **Race**  White  Black  Asian  Latino | 5 (62.5%)  2 (25%)  0 (0%)  1 (12.5%) | 3 (27.3%)  2 (18.2%)  4 (36.4%)  2 (18.2%) | 3 (42.9%)  2 (28.6%)  1 (14.3%)  1 (14.3%) | 4 (66.7%)  0 (0%)  0 (0%)  2 (33.3) |
| **Vaccination status** | 6 (75%) | 3 (27.3%) | 1 (14.3%) | 0 (0%) |
| **Charlson Comorbidity Score, median (25^th^-75^th^ percentiles)** | 0 (0-1) | 0 (0-2) | 1 (0-2) | 1 (0-2.2) |
| **Time of sample after symptoms onset (days), median (25^th^-75^th^ percentiles)** | 81 (78-91) | 84 (78-86) | 83 (78-85) | 97 (69-107) |
| **NIAID Highest Ordinal scale**  4 (no oxygen)  5 (low-flow oxygen  6 (high-flow oxygen) | 8 (100%)  0 (0%)  0 (0%) | 9 (81.8%)  0 (0%)  2 (18.2%) | 2 (28.6%)  4 (57.1%)  1 (14.3%) | 2 (33.3%)  0 (0%)  4 (66.7%) |
| **NIAID Ordinal scale at time of sample**  4 (no oxygen)  5 (low-flow oxygen  6 (high-flow oxygen) | 8 (100%)  0 (0%)  0 (0%) | 10 (90.9%)  1 (9.1%)  0 (0%) | 6 (85.7%)  1 (14.3%)  0 (0%) | 3 (50%)  2 (33.3%)  1 (16.7%) |

**e-Table 5. Differentially expressed proteins in vaccinated Omicron patients versus healthy controls and pre-Omicron patients in the acute and convalescent phases**

|  | **Comparisons** | **Differentially**  **Overexpressed Proteins** | **Differentially**  **Underexpressed Proteins** |
| --- | --- | --- | --- |
| **Acute** | Omicron (n=26)  Vs  Healthy Controls (n=20) | 52 | 781 |
|  | Omicron (n=26)  Vs  Pre-Omicron (n=14) | 0 | 0 |
| **Convalescent** | Omicron (n=20)  Vs  Healthy Controls (n=20) | 45 | 764 |
|  | Omicron (n=20)  Vs  Pre-Omicron (n=10) | 0 | 0 |

**Figures**

**Supplementary Figure S1**

**(a)**

**(b)**

**Figure S1.** (a) Metascape Enrichment Pathway analysis of the overexpressed proteins comparing all vaccinated SARS-CoV-2 patients versus healthy controls, and vaccinated SARS-CoV-2 patients with samples obtained within 30 days from symptoms onset versus healthy controls. Vax – vaccinated. (b) Metascape Enrichment Pathway analysis of the overexpressed proteins comparing all unvaccinated SARS-CoV-2 patients versus healthy controls, and unvaccinated SARS-CoV-2 patients with samples obtained within 30 days from symptoms onset versus healthy controls. Unvax – unvaccinated. Gradation of colors reflect the -log 10 (p) value which indicates the statistically enriched terms using Metascape.

**Supplementary Figure S2**

(a) (b)

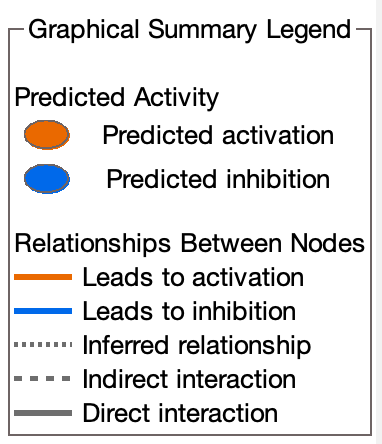


**Figure S2.** Graphical summary of differentially overexpressed proteins and pathways during acute SARS-CoV-2 infection using Ingenuity Pathway Analysis in (a) unvaccinated, and (b) vaccinated patients.

**Supplementary Figure S3**

(a) (b)


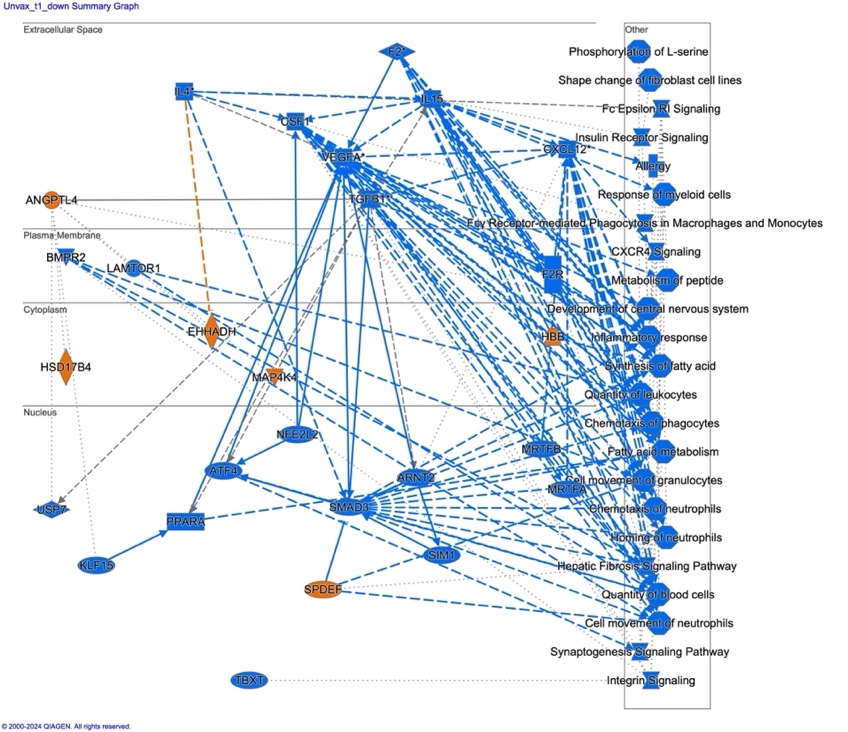

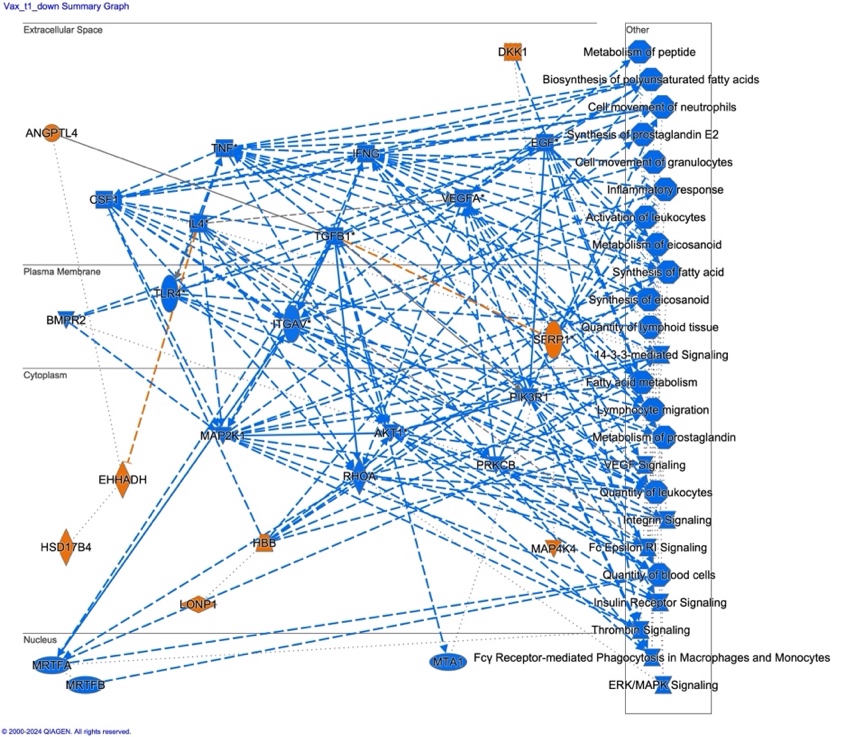

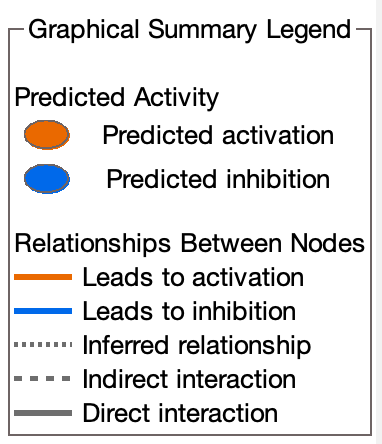


**Figure S3.** Graphical summary of differentially underexpressed proteins and pathways in unvaccinated patients during acute SARS-CoV-2 using Ingenuity Pathway Analysis in (a) unvaccinated, and (b) vaccinated patients.

**Supplementary Figure S4**


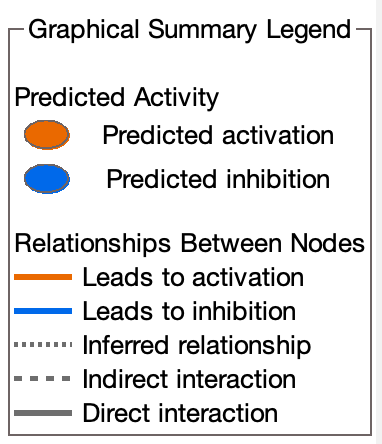


**Figure S4.** Graphical summary of differentially overexpressed proteins and pathways in unvaccinated patients in the post-infection phase at 3 months after SARS-CoV-2 infection using Ingenuity Pathway Analysis.

**Supplementary Figure S5**

(a) (b)

**
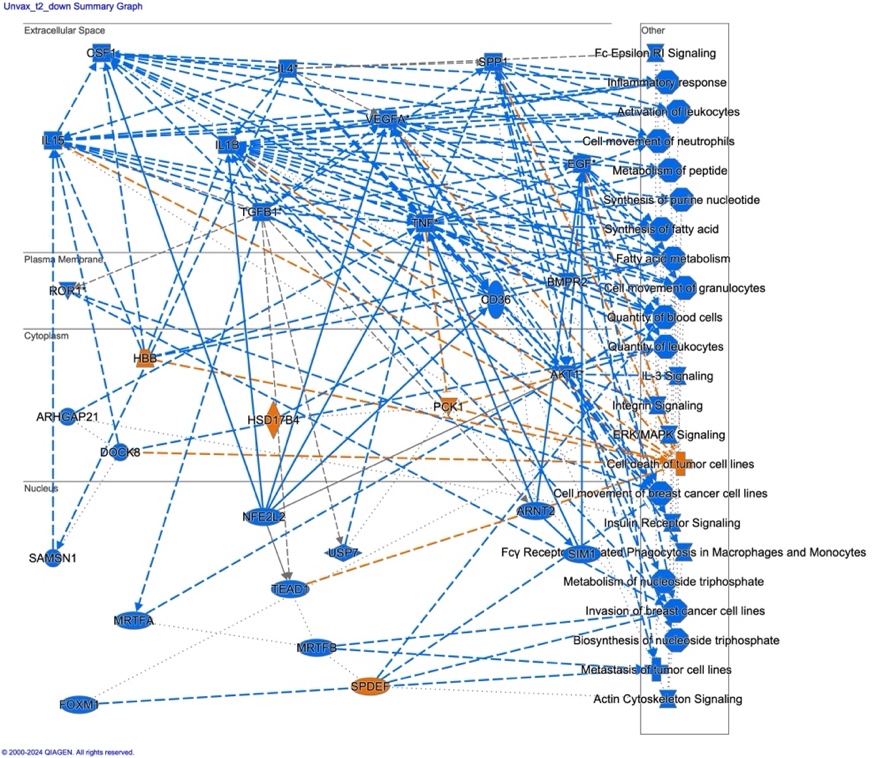

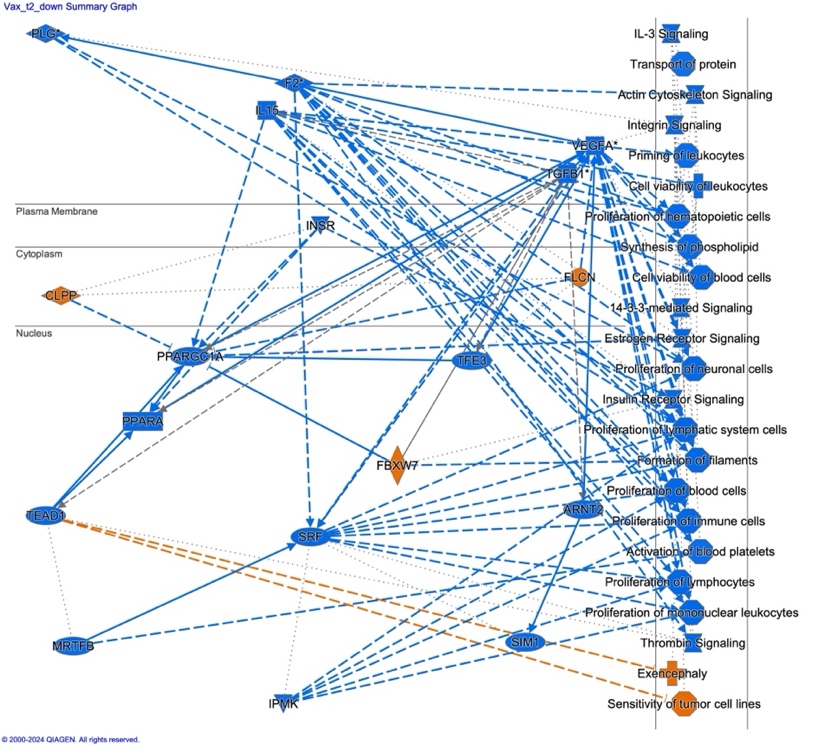
**
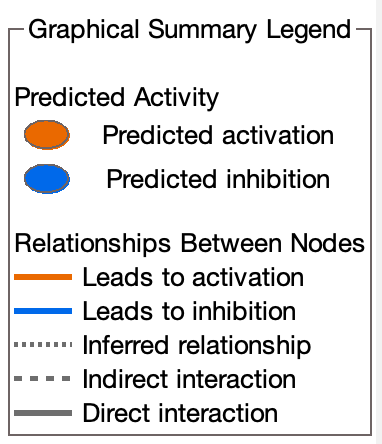


**Figure S5.** Graphical summary of differentially underexpressed proteins and pathways in the post-infection phase at 3 months after SARS-CoV-2 infection using Ingenuity pathway analysis in (a) unvaccinated, and (b) vaccinated patients.

**Supplementary Figure S6**


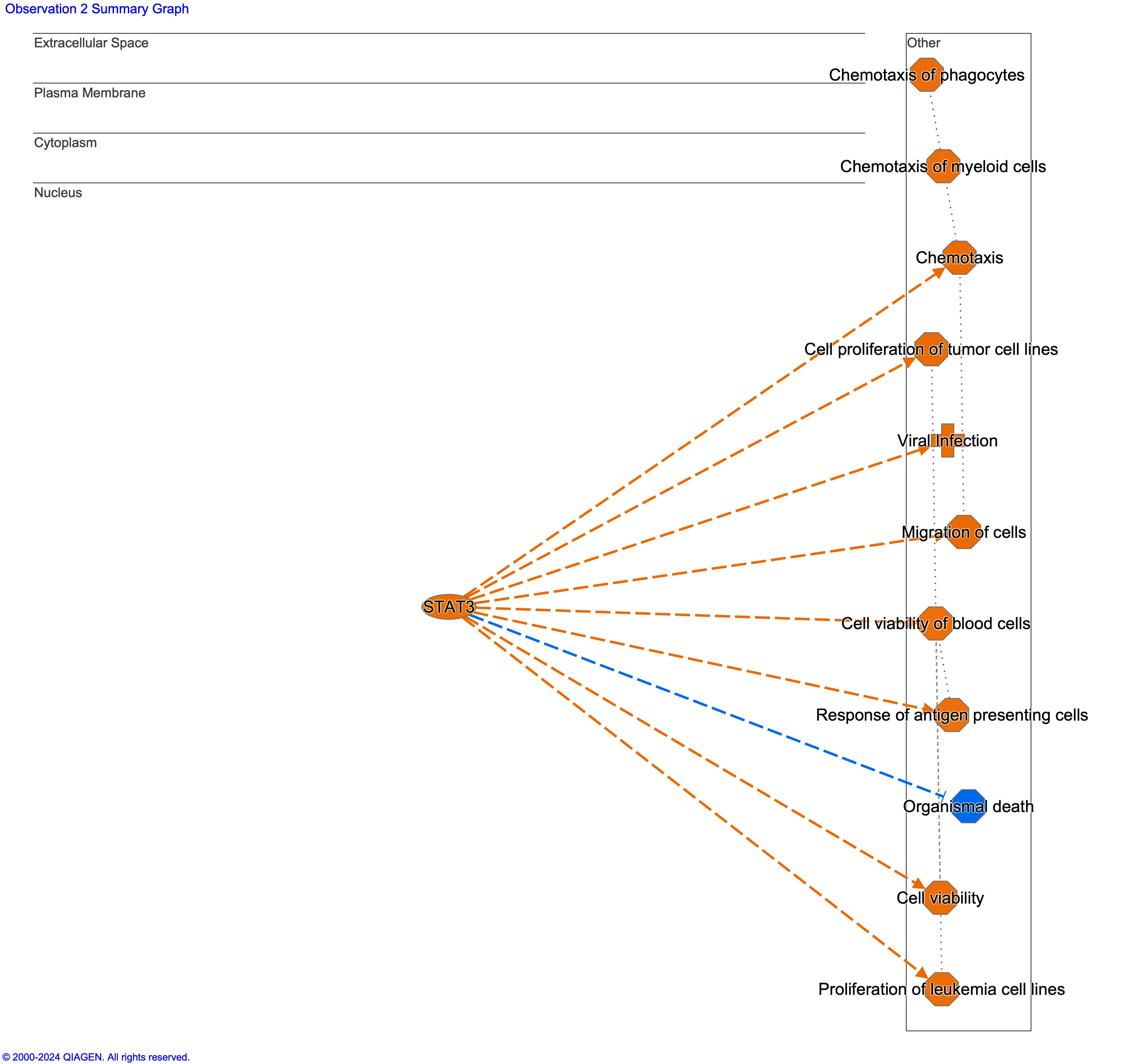

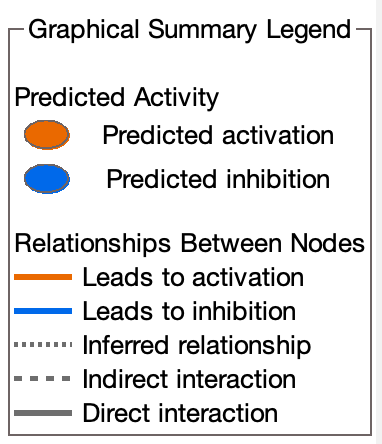


**Figure S6.** Graphical summary of differentially overexpressed proteins and pathways in unvaccinated patients in the post-infection phase at 10 months after SARS-CoV-2 infection using Ingenuity pathway analysis.

**Supplementary Figure S7**

**
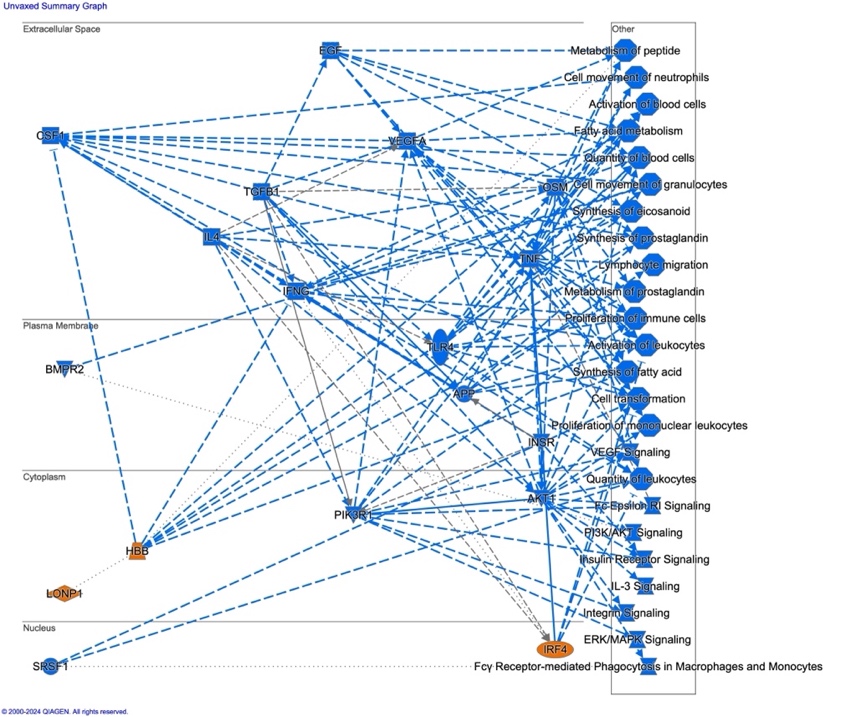

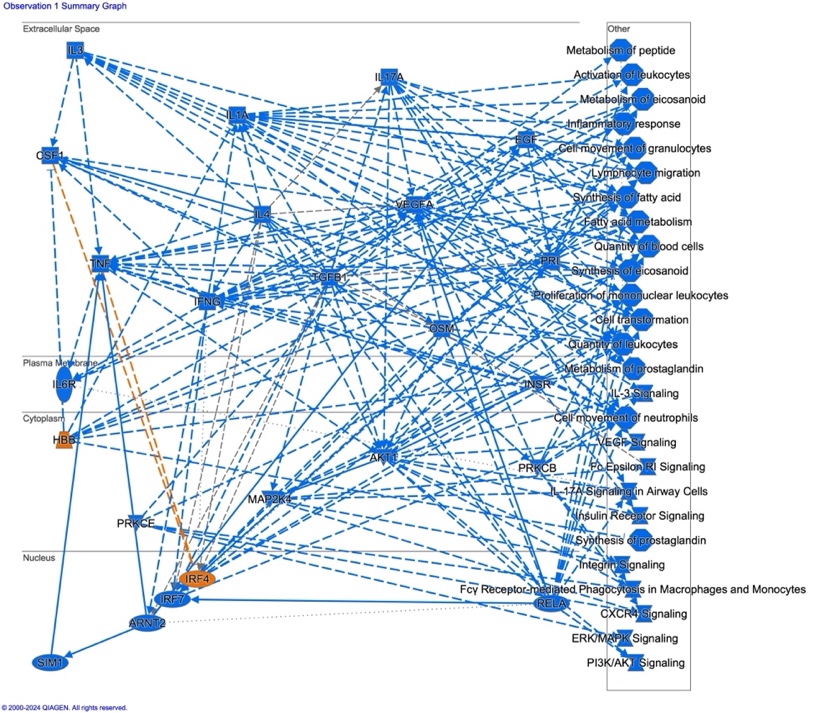
**
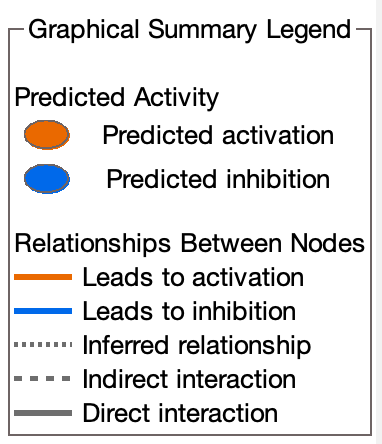


**Figure S7.** Graphical summary of differentially underexpressed proteins in the post-infection phase at 10 months using Ingenuity pathway analysis in (a) unvaccinated, and (b) vaccinated patients.

**Supplementary Figure S8**


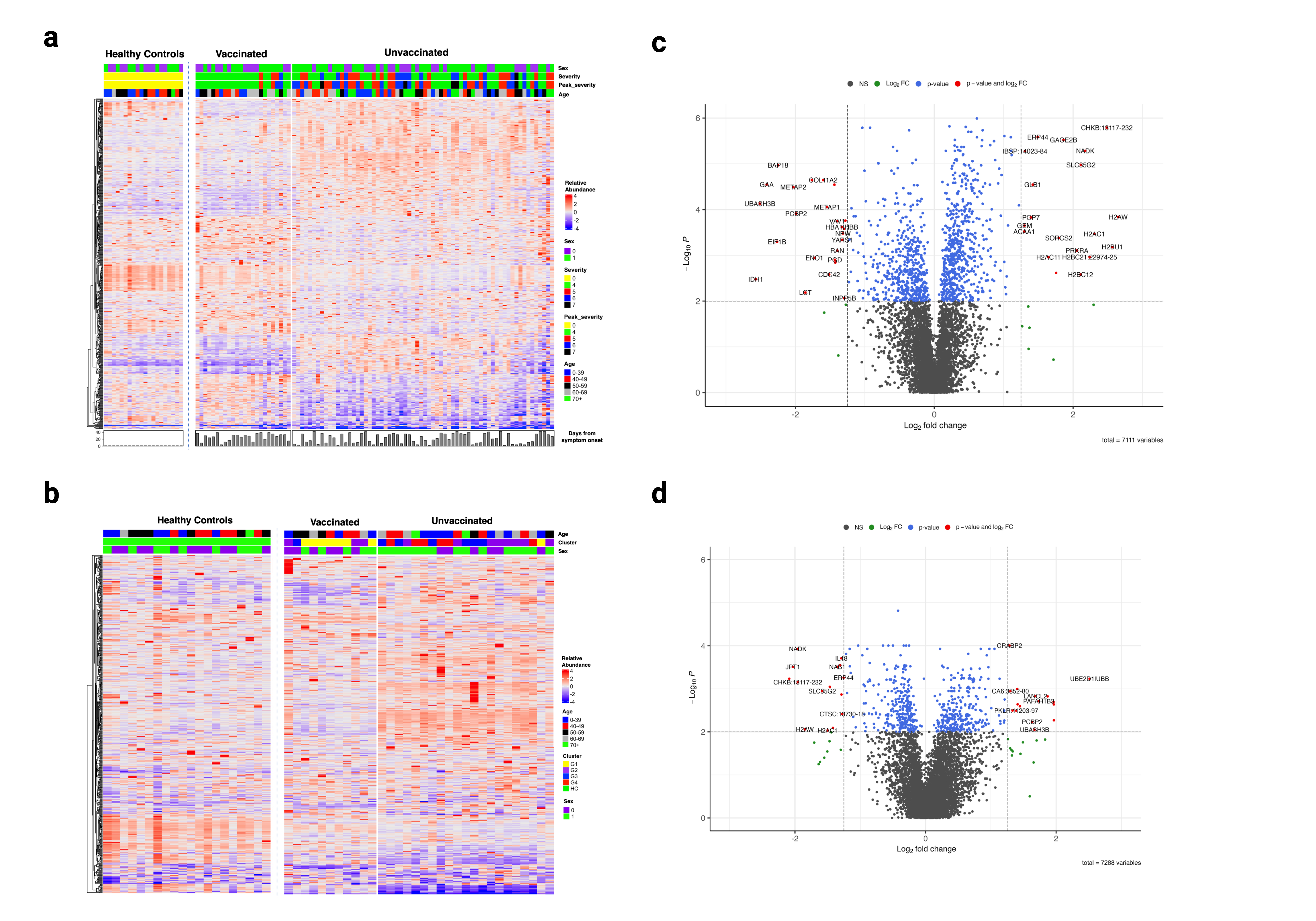


**Figure S8.** **Plasma proteome comparing directly vaccinated and unvaccinated SARS-CoV-2 patients.** *(a and b) Heatmap showing differentially expressed proteins (DEP) between unvaccinated and vaccinated SARS-CoV-2 patients at (a) acute (66 unvaccinated and 24 vaccinated) and (b) three months post-infection (22 unvaccinated ,10 vaccinated). Healthy controls are shown for reference only. (b and c) Volcano plot of DEP in plasma proteome comparing b) unvaccinated and vaccinated SARS-CoV-2 patients at three months post-infection and c) at ten months post-infection . Horizontal dashed line denotes a cutoff of 0.01 for the FDR corrected p value after age, sex, and race adjustments. Vertical dashed line denotes a cutoff of 1.25 for the fold change.*

**Supplementary Figure S**
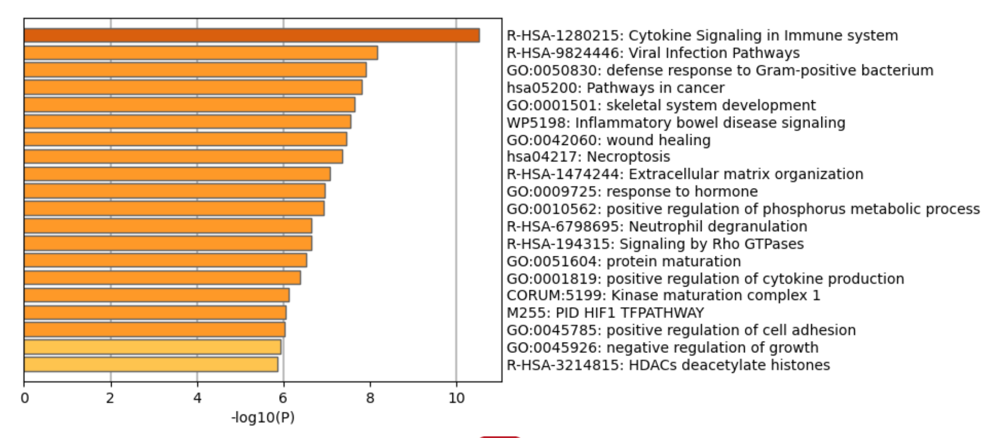
**9**

**Figure S9.** Metascape Enrichment Pathway analysis of the overexpressed proteins comparing unvaccinated vs vaccinated SARS-CoV-2 patients in the acute phase. Gradation of colors reflect the -log 10 (p) value which indicates the statistically enriched terms using Metascape.

**Supplementary Figure S10**


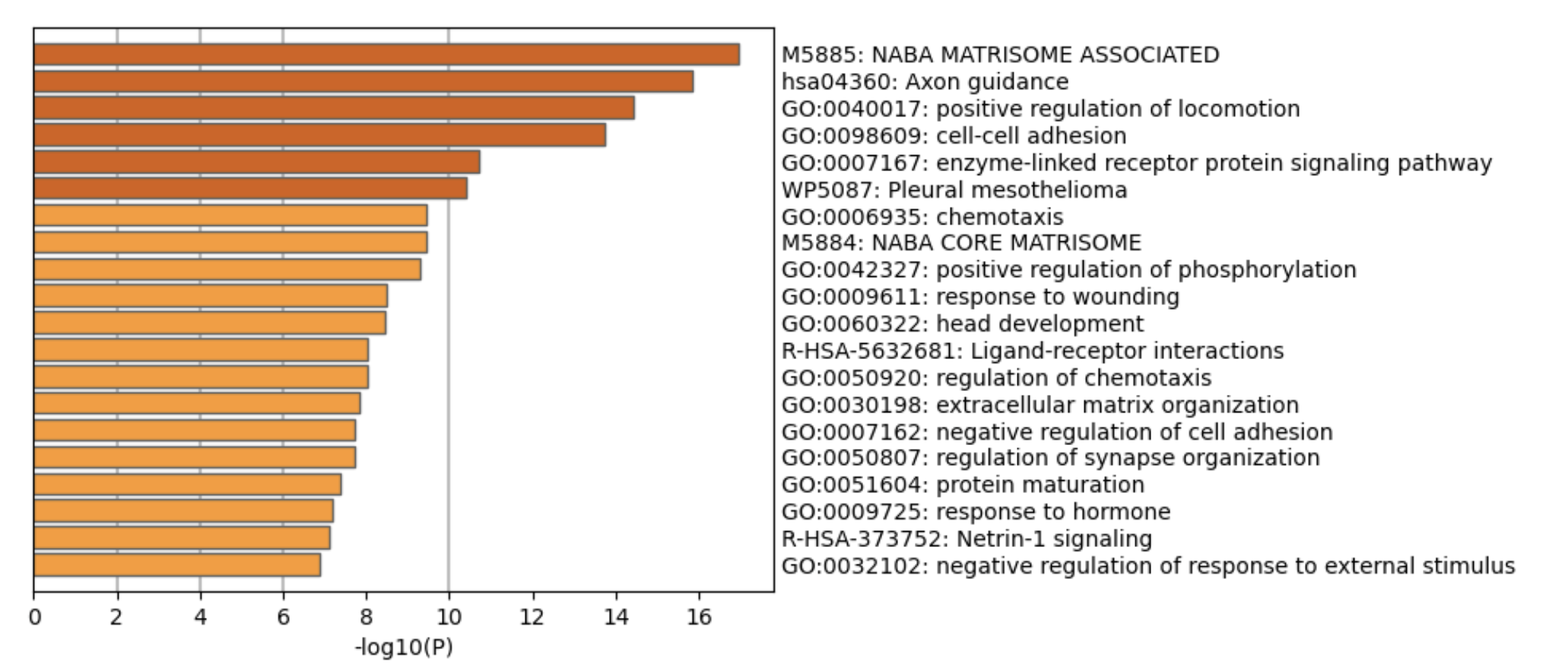


**Figure S10**. Metascape Enrichment Pathway analysis of the underexpressed proteins comparing unvaccinated vs vaccinated SARS-CoV-2 patients in the acute phase. Gradation of colors reflect the -log 10 (p) value which indicates the statistically enriched terms using Metascape.

**Supplementary Figure S11**


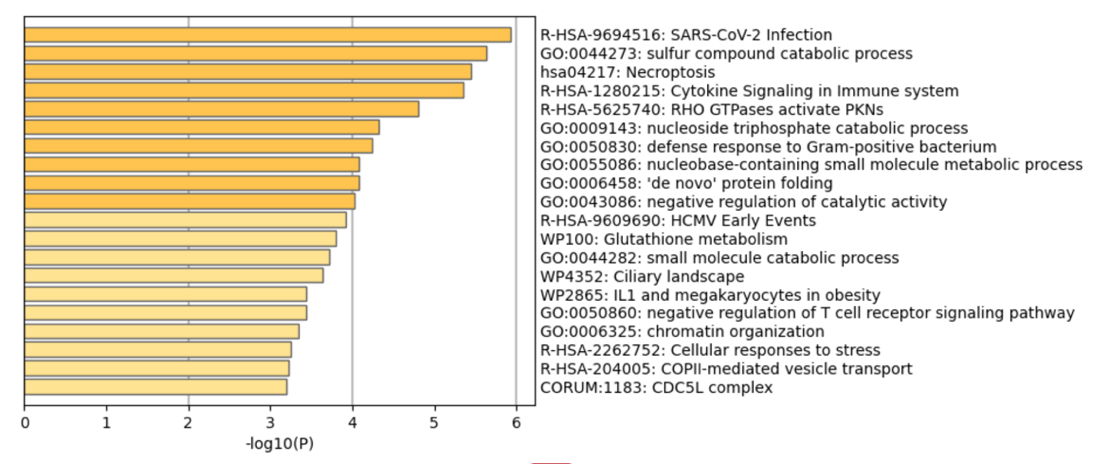


**Figure S11.** Metascape Enrichment Pathway analysis of the overexpressed proteins comparing unvaccinated vs vaccinated SARS-CoV-2 patients in the 3-month post-infection phase. Gradation of colors reflect the -log 10 (p) value which indicates the statistically enriched terms using Metascape.

**Supplementary Figure S12**


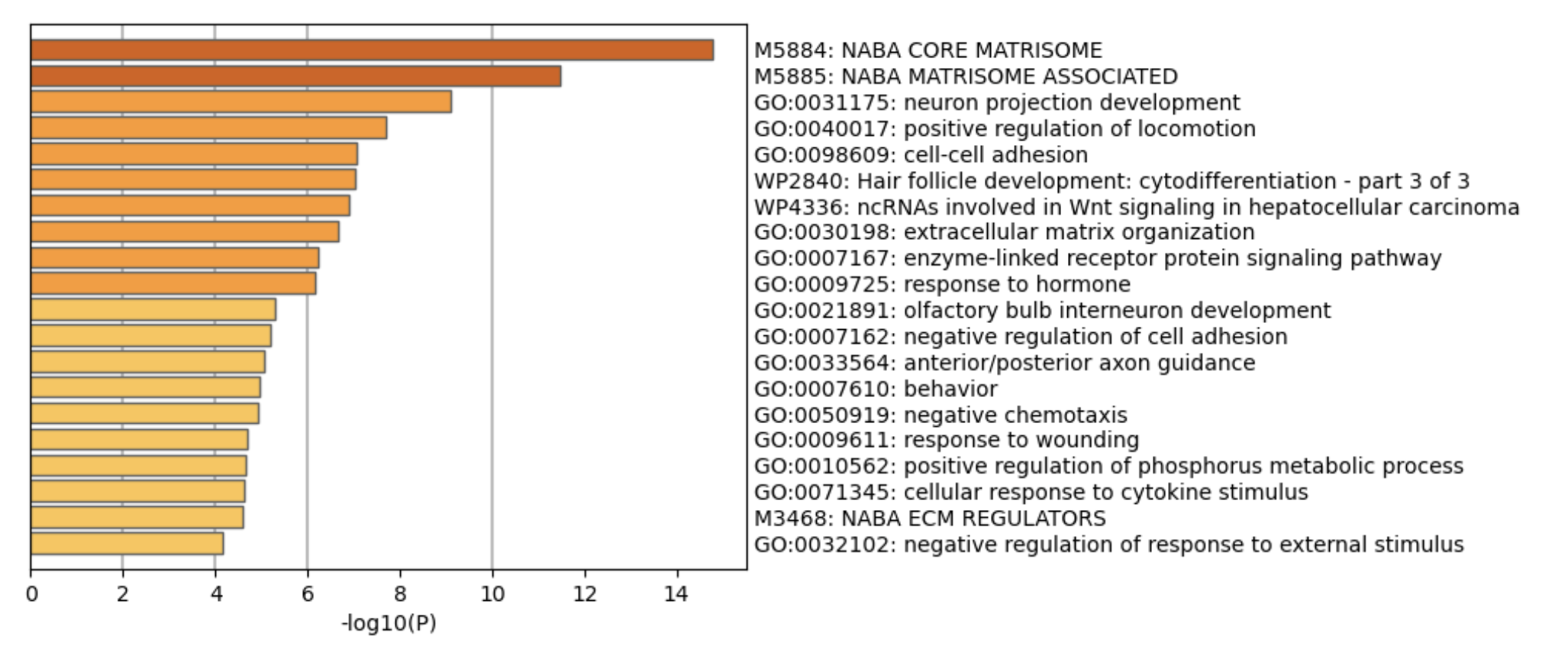


**Figure S12.** Metascape Enrichment Pathway analysis of the underexpressed proteins comparing unvaccinated vs vaccinated SARS-CoV-2 patients in the 3-month post-infection phase. Gradation of colors reflect the -log 10 (p) value which indicates the statistically enriched terms using Metascape.

**Supplementary Figure S13**

**Figure S13.** UMAP of post-infection samples of 4 SARS-CoV-2 group identified by hierarchical clustering at 3 months post-infection. (a) Uniform manifold approximation and projection (UMAP) of acute changes in plasma proteomics from 90 SARS-CoV-2 persons and 20 healthy controls. Circles - unvaccinated, triangles - vaccinated, and squares - healthy controls. Yellow – G1, purple – G2, blue – G3, and red – G4.

**Supplementary Figure S14**

**Figure S14.** Heatmap of differentially expressed proteins between each SARS-CoV-2 group, identified via hierarchical clustering, and HC in the 3-month post-infection phase

**Supplementary Figure S15**

**Figure S15.** Random forest analysis to predict cardiopulmonary symptoms, musculoskeletal symptoms, and neurological symptoms at 10 months from infection. The variables with larger mean decrease in Gini index had greater variable importance in symptom predictions.

**Supplementary Figure S16**

**(a) (b)**


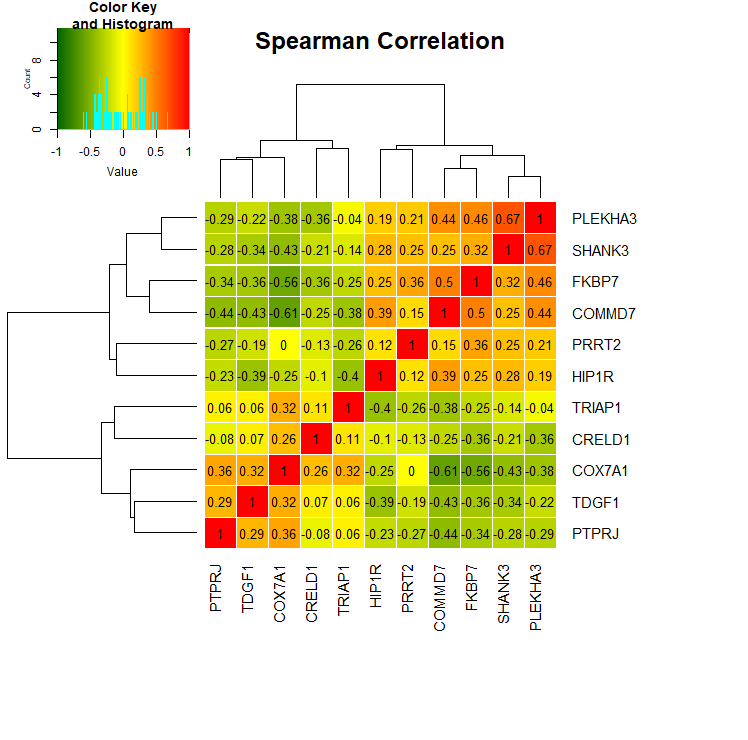

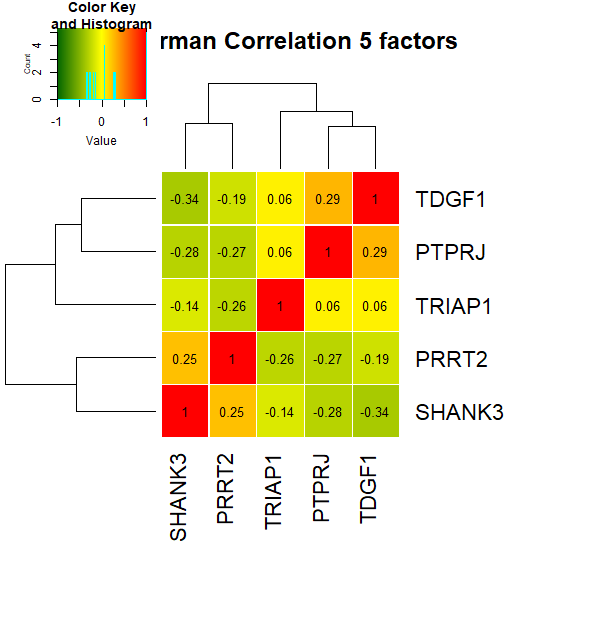


**(c) (d)**


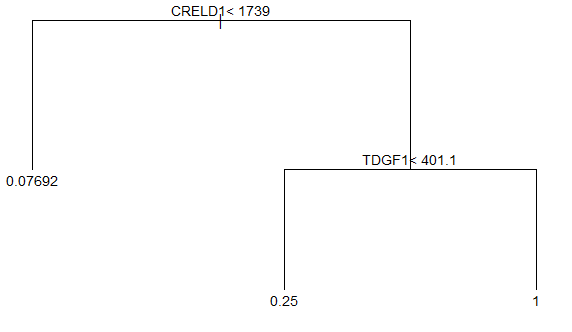

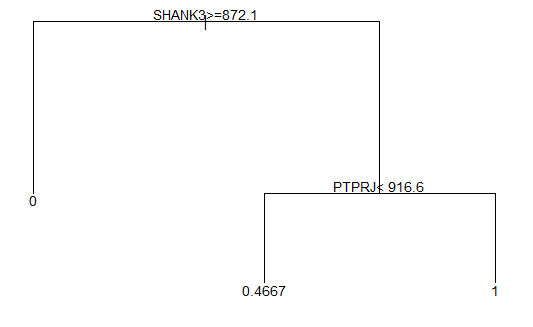


**Figure S16.** Prediction Tree identified via Random Forest analysis based on the selection of the most relevant proteins among the top 30 to build up a model to predict long SARS-CoV-2 symptoms at ten months. Spearman correlation using 11 (a) and 5 (b) proteins. Classification And Regression Tree (CART) model based on 11 (c) and 5 (d) proteins.

**Supplementary Figure S17**

**A**

**
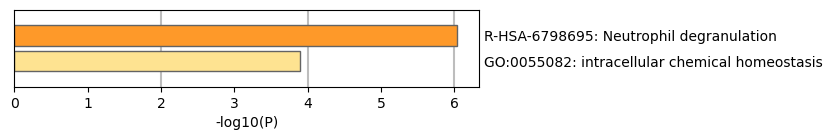
**

**B**

**
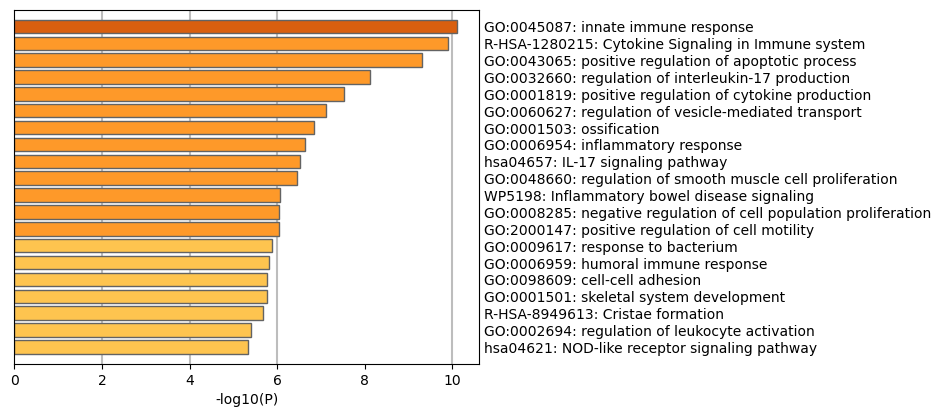
**

**C**

**
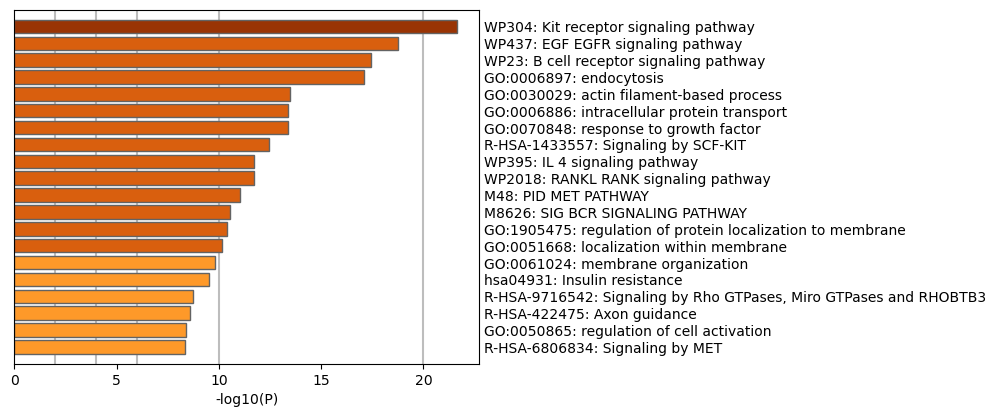
**

**Figure S17.** Metascape Enrichment Pathway analysis of proteins that significantly changed over time in unvaccinated SARS-CoV-2 patients. (a) Cluster 1 included 6 proteins that decreased from acute to 3-month follow-up. (b) Cluster 2 included 384 proteins that decreased from 3-month follow-up to 10-month follow-up. (c) Cluster 3 included 212 proteins that increased from 3-month follow-up to 10-month follow-up. Gradation of colors reflect the -log 10 (p) value which indicates the statistically enriched terms using Metascape.

**Supplementary Figure S18**

**Figure S18**. (B) Heatmap of differentially expressed proteins (DEP) in vaccinated Omicron (Om) and Pre-Omicron (Pre-Om) SARS-CoV-2 patients compared to healthy controls. Each row represents a single protein; each column represents a patient. A: Acute; C: Convalescent.

**Supplementary Figure S19**

**Figure S19.** Metascape Enrichment Pathway analysis of (A and B) overexpressed proteins in vaccinated Omicron SARS-CoV-2 patients vs healthy controls in the acute (A) and convalescence (B) phases; and of underexpressed proteins in vaccinated Omicron SARS-CoV-2 vs healthy controls in the acute (C) and convalescence (D) phases. Gradation of colors in e and f reflect the -log 10 (p) value which indicates the statistically enriched terms using Metascape.

**References**

1. Huapaya JA*, et al.* Vaccination Ameliorates Cellular Inflammatory Responses in SARS-CoV-2 Breakthrough Infections. *J Infect Dis* **228**, 46-58 (2023).
